# Maintenance of bone health by osteoblasts in the absence of osteocytes: comparative analysis of gene expression in medaka and zebrafish vertebrae

**DOI:** 10.1016/j.bonr.2026.101953

**Published:** 2026-09-30

**Authors:** S.N. Jayash, D.R. Dunbar, S. Guizard, J. Furniss, R. Ruiz Daniels, A.A. Pitsillides, K.A. Staines, D. Robledo, C. Farquharson, L.A. Stephen

**Affiliations:** a The Roslin Institute and Royal (Dick) School of Veterinary Studies, University of Edinburgh, UK; b Institute of Aquaculture, University of Stirling, UK; c Comparative Biomedical Sciences, The Royal Veterinary College, Royal College Street, London, UK; d Centre for Lifelong Health, School of Applied Sciences, University of Brighton, UK

**Keywords:** Osteocyte, Osteoblast, Bone, Medaka, Zebrafish, snRNA-seq

## Abstract

Osteocytes are regarded as *the* strain-sensing bone cells; their capacity to sense and convert mechanical loads into biological signals underpins our current understanding of how bone is (re)modelled to normalise local strains. However, most extant fish are anosteocytic and lack osteocytes, yet their bone structure and (re)modelling abilities remain unimpeded, sitting uncomfortably with this paradigm. We propose that alternative, non-osteocytic, mechano-sensing mechanisms are present in anosteocytic fish bones. We examined gene expression in anosteocytic medaka bone and compared it with osteocytic zebrafish bone to identify alternative mechano-sensory modes. In situ hybridization found *sost* and *fgf23* to be expressed by osteocytes in zebrafish, whereas in medaka bone, strong expression of both genes was observed in osteoblasts. snRNA-seq analysis indicated 12 distinct transcriptional clusters in medaka and zebrafish bone, each corresponding to a specific cell type. One such cluster included a concentration of osteoblast- and osteocyte-associated genes, including signature osteocyte genes in both species. Although many were expressed at very low levels by a small proportion of nuclei, expression of *irx5a, dkk3b, emilin2a, cfap70 and susd5* was higher in medaka, while *fgf23*, fbln7, *prr15lb, sost, seema3a and ngef* expression was higher in zebrafish. We conclude that osteocyte functions are likely evolutionarily transferred to osteoblasts in a model whereby osteoblasts may have developed osteocytic potential at an earlier stage in the osteogenic lineage than has previously been identified.

## Highlights

• Anosteocytic fish maintain bone modelling without osteocytes.
• snRNA-seq identifies osteoblast and osteocytes genes markers in medaka and zebrafish.
• Medaka osteoblasts express key osteocyte markers (sost, fgf23).
• Osteoblasts may assume osteocyte-like functions in anosteocytic bone.

## 1 Introduction

Structural adaptation to load-bearing cues (mechano-adaptation) is critical in the attainment and maintenance of bone mass and architecture to ensure sufficient strength (Lanyon, 1993; Burr et al., 2002). The vital roles of this mechano-adaptation are demonstrated in clinical studies, particularly in high-impact sports (Spector et al., 2009; Shimano and Volpon, 2009) and bed rest (Spector et al., 2009). Animal studies exploit externally applied load or unloading (Shimano and Volpon, 2009; Hanson et al., 2005; Salmon et al., 2023) while in vitro studies examine strain or fluid flow applied to bone cells to understand the cellular and molecular processes underlying the role of osteocytes (Liu et al., 2010; Ponik et al., 2007; Suswillo et al., 2017). Bone adaptation to mechanical loading is widely attributed to osteocytes, which function as embedded mechano-sensors within the mineralized matrix of trabecular and cortical bone. The extensive osteocyte dendritic morphology creates multicellular networks (lacuna-canalicular) permeating bone's mineral matrix, permitting direct contact with neighbouring osteocytes, surface osteoblasts and osteoclasts in the bone marrow (Dallas et al., 2013). Present in all tetrapods, osteocytes have now emerged as versatile orchestrators of bone (re)modelling (Dallas et al., 2013). How osteocytes sense the mechanical milieu is uncertain, but their cell body, dendritic processes or bending of cilia may work alone or in combination to sense and transmit mechanical stimuli (Bonewald, 2006). Ultimately, transduction of these stimuli into biological signals directs bone formation and bone resorption.

Given that osteocytes and their network of interconnected processes serve pivotal ‘strain gauging’ roles, it is intriguing that the entire neoteleost (evolutionary - advanced and extant fish) skeleton is devoid of osteocytes and their associated lacuna-canalicular network (Weiss and Watabe, 1979; Ekanayake and Hall, 1988a; Shahar and Dean, 2013; Davesne et al., 2019; Ofer et al., 2019a); this is at odds with the accepted doctrine of osteocyte function. The observation that osteocytes are absent in neoteleost bone was first made in 1859 by Albert von Kölliker, but it was not until ∼100 years later that this anosteocytic state emerged as a derived trait (Moss, 1961a). Indeed, it is now proposed that the loss of osteocytes in neoteleosts (>50% of extant fish) confers a skeletal advantage over less advanced fish that possess osteocytes (Moss, 1961a). The exact character of this putative advantage is, however, completely undefined. The advantage of this evolutionary loss of osteocytes in neoteleosts must in some way explain how the mechanical properties and mineral density of osteocytic and anosteocytic bones can be so similar, despite this fundamental difference in their resident cell population (Ofer et al., 2019a; Horton and Summers, 2009; Ofer et al., 2021). This is especially pertinent given that the presence/absence of osteocytes is the only major difference between the bones of these two clades that are separated by a large phylogenetic difference of 115–200 million years (Wittbrodt et al., 2002; Furutani-Seiki and Wittbrodt, 2004).

These findings challenge the central axiom that osteocytes alone are responsible for strain-sensing and for coordinating mechano-adaptive (re)modelling. Anosteocytic bone adapts to applied mechanical loads and undergoes modelling despite the complete absence of osteocytes (Ofer et al., 2019a; Tauer et al., 2024; Atkins et al., 2015a). We must therefore conclude that anosteocytic bone possesses alternative, non-osteocytic, mechanisms for both the detection of load-induced mechanical strain and for the cellular cross-talk needed to coordinate bone (re)modelling accordingly. Although the alternative mechano-sensing mechanisms used to regulate (re)modelling without osteocytes are unclear, it is known that swim training causes a reduction in *sost* expression by osteoblasts, chondrocytes and chordoblasts of the medaka vertebrae leading to increased bone formation (Ofer et al., 2019a). Furthermore, a more recent RNAseq study identified changes in the expression of genes linked to the extracellular matrix, skeletal development and matrix mineralization in spines of osteocytic and anosteocytic fish subjected to a brief swim training session (Tauer et al., 2024).

These studies unmistakably show that anosteocytic bone can undergo adaptive modelling in response to mechanical strain, potentially transferring osteocyte-associated functions to other skeletal cells. For this to be the case, we may expect that the osteocyte “gene signature” would be found in non-osteocytes, but to date a full transcriptomic analysis at single-cell resolution to identify these alternative cell(s) has not been reported. Therefore, in this study we compared the bone of non-swim-trained osteocyte-containing zebrafish to that of the anosteocytic medaka using single-nucleus RNA sequencing (snRNA-seq) and in situ hybridization. This combined approach provides novel insights into how bone health is maintained in the absence of osteocytes.

## 2 Materials and methods

### 2.1 Fish

Wildtype zebrafish (*Danio rerio*) and medaka (*Oryzias latipes*) were bred and maintained in tanks at the BVS Aquatics Unit at Queens Medical Research Institute, University of Edinburgh. The unit is a home office designated facility, and all fish were maintained in accordance with the Animals (Scientific Procedures) Act 1986 and directive 2010/63EU. Six-month-old adult fish were euthanised using an overdose of anaesthetic (MS-222/tricaine), followed by confirmation of death by destruction of the brain, in accordance with the UK Animals (Scientific Procedures) Act 1986. The head, scales and as much soft tissue as possible were removed; the remaining vertebrae were snap-frozen in liquid nitrogen and stored at −80 °C for further processing. The number of fish used in each experiment is summarized in Table 1. To minimise potential circadian effects on gene expression, fish from all experimental groups were euthanized in the morning within a 2-h window, and tissue collection was performed immediately thereafter under identical conditions.

**Table 1:** Summary of experimental cohorts.

Experiment	Zebrafish	Medaka
H&E histology	*n* = 3 fish	n = 3 fish
RNA-FISH	n = 3 fish	n = 3 fish
snRNA-seq	2 samples (2 individual fish)	2 pooled samples (4–5 fish per pool; total 8–10 fish)
qPCR	3 samples (3 individual fish)	3 pooled samples (2–3 fish per pool; total 6–9 fish)

### 2.2 Haematoxylin and eosin staining

Sections of the caudal vertebral region, including surrounding soft tissues from six-month-old zebrafish and medaka were fixed in 4% paraformaldehyde (PFA) for 24 h at 4 °C. Fixed whole fish were decalcified in 14% ethylenediaminetetraacetic acid for 1 week, followed by washing in running water overnight. Tissues were then processed and embedded in paraffin wax according to standard histological procedures. Caudal vertebrae were sectioned at a thickness of 5 μm to obtain transverse sections through the vertebral bone surrounding the notochord (Ekanayake and Hall, 1987). Sections were mounted on glass slides and stained with haematoxylin and eosin (H&E) following standard protocols (Stephen et al., 2015).

### 2.3 HCR™ RNA fluorescence in situ hybridization (RNA-FISH)

Whole-mount RNA fluorescence in situ hybridization was performed using the HCR™ RNA-FISH protocol (v3.0; Molecular Instruments, Los Angeles, USA) according to the manufacturer's instructions with minor adaptations for sections of the caudal vertebral region, including surrounding soft tissues. Briefly, vertebrae were fixed in 4% PFA overnight at 4 °C, followed by washing in phosphate-buffered saline containing 0.1% Tween-20 (PBST). Vertebrae were dehydrated through a graded methanol series and stored in 100% methanol at −20 °C until further processing. Prior to hybridization, vertebrae were rehydrated through a reverse methanol series to PBST and permeabilized using proteinase K treatment (120 μg/mL, Promega UK, Ltd., Southampton, UK) for 3 h. Vertebrae were then post-fixed in 4% PFA, washed thoroughly in PBST, and pre-hybridized in HCR probe hybridization buffer at 37 °C.

Custom HCR probe sets specific to target transcripts were hybridized overnight at 37 °C (Supplementary Table 1). Following hybridization, vertebrae were subjected to a series of washes using HCR probe wash buffer to remove unbound probes. Amplification was performed by incubating samples with fluorophore-labelled HCR hairpins, which had been snap-cooled according to the manufacturer's protocol, in amplification buffer overnight at room temperature in the dark. After amplification, samples were washed extensively in 5% saline‑sodium citrate buffer with Tween-20 (SSCT) to reduce background fluorescence. For sectioning, samples were embedded in optimal cutting temperature (OCT) compound and cryo-sectioned at a thickness of 20 μm using a Leica CM1950 Cryostat (Leica Biosystems, Wetzlar, Germany). Sections were mounted onto glass slides and air-dried for 3–4 h at room temperature to ensure proper adhesion. Slides were either processed immediately for staining or stored at −20 °C until further use. Nuclei were counterstained with DAPI (AAT Bioquest, USA) and samples were cleared and mounted for imaging. Fluorescent signals were visualized using a Zeiss LSM 880 confocal microscope equipped with an Airyscan detector (Carl Zeiss, Germany). Image acquisition was performed using ZEN software (Zeiss) and ImageJ (FIJI; National Institutes of Health).

### 2.4 Nuclei isolation from bone tissue for single-nucleus RNA-sequencing (snRNA-seq)

For extraction of nuclei, all steps were carried out on ice, unless otherwise stated. Frozen vertebrae were mechanically dissociated in ice-cold TST buffer, consisting of 1× Salt-Tris (ST) buffer supplemented with 0.03% Tween-20 (Sigma-Aldrich, UK) and 0.01% BSA. The ST buffer was prepared using NaCl and Tris-HCl (pH 7.5) (both from Thermo Fisher Scientific, UK), CaCl₂ (Sigma-Aldrich, UK), and MgCl₂ (Merck, Germany). For RNA-seq runs, RNase inhibitor (Merck, Germany) was added to the buffer at a final concentration of 40 U/mL.

Tissue fragments were minced on ice using spring scissors, followed by gentle pipetting for 10 min to aid mechanical disruption. The lysate was filtered through a 40 μm cell strainer (Sigma-Aldrich, UK) and washed with additional buffer to maximize recovery. The suspension volume was adjusted to 5 mL with 1× ST buffer and centrifuged at 500 ×*g* for 5 min at 4 °C. The nuclear pellet was resuspended in fresh buffer and passed through a second cell strainer (typically 20 μm, pluriSelect, Germany) to improve purity. Nuclei were counted and assessed for quality using a Millicell® disposable hemocytometer (Merck, Germany) prior to downstream library preparation. For downstream nuclei fixation, a minimum of 500,000 nuclei per sample was required. Therefore, zebrafish samples (*n* = 2) were processed individually, but each medaka sample (*n* = 2) was pooled from 4 to 5 fish to ensure sufficient nuclei yield for successful processing (Ruiz Daniels et al., 2023).

### 2.5 Nuclei fixation

Nuclei fixation was performed using the Evercode™ Nuclei Fixation Kit v2 (Parse Biosciences, USA) according to the manufacturer's instructions. Briefly, two single-nuclei suspensions, each containing 500,000 nuclei, were prepared from each sample and kept on ice. Nuclei were transferred into a 15 mL polypropylene tube. After centrifugation (200 ×*g*, 10 min, 4 °C), the pellet was resuspended in cold nuclei buffer with bovine serum albumin (BSA) and filtered through a 40 μm strainer. Fixation was initiated by adding nuclei fixation solution, followed by a brief incubation on ice and the addition of permeabilization solution. After further incubation, fixation was quenched with nuclei neutralization buffer. The nuclei were pelleted again and resuspended in nuclei buffer containing RNase inhibitor. A second filtration was performed, followed by a stepwise addition of dimethyl sulfoxide for cryoprotection. Fixed nuclei were counted, aliquoted (if necessary), and stored at −80 °C in a controlled-rate freezing container until library preparation. Examination of the nuclei following fixation confirmed their morphological integrity and the absence of visible aggregation or lysis.

### 2.6 Library preparation

#### 2.6.1 Parse biosciences Evercode™ protocol

Single-nucleus RNA-seq libraries were prepared using the Evercode™ Whole Transcriptome v2 protocol (Parse Biosciences, USA), following the manufacturer's instructions. Fixed nuclei were processed through a multi-step barcoding and library preparation workflow, enabling high-throughput and combinatorial indexing of individual nuclei.

#### 2.6.2 Barcoding and sublibrary generation

Barcoding was performed through a three-step combinatorial indexing strategy. In the first round, the fixed and permeabilized nuclei were distributed into a 48-well plate, and RNA was reverse transcribed using oligodT and random hexamer primers with a well-specific barcode associated with specific samples. After cDNA synthesis, nuclei were pooled and randomly distributed onto a 96-well plate, where a second well-specific barcode was ligated to the first barcode. This process was repeated to add a third barcode containing a biotin molecule. Nuclei were then pooled a final time, counted and evenly redistributed into 8 sub-libraries and subsequently lysed.

#### 2.6.3 cDNA amplification

Barcoded cDNA was enriched from the lysate by binding to streptavidin-magnetic beads, followed by a template switch step to complete full-length cDNA synthesis. Amplification of cDNA was performed using PCR, and amplified products were purified with SPRI bead clean-up to remove excess primers and reagents.

#### 2.6.4 Library construction for sequencing

Amplified cDNA underwent fragmentation, end-repair, and A-tailing, followed by a double-sided SPRI bead selection to ensure proper fragment size distribution. Sequencing adapters were ligated, and adapter-ligated products were purified. Indexing PCR was performed to generate sub-library-specific indices, and final double-sided SPRI selection was applied to obtain high-quality, sequencing-ready libraries.

#### 2.6.5 Sequencing and data generation

Final libraries were quality-checked and sequenced by Novogene (UK). The libraries were submitted as pre-made libraries and sequenced using lane-based paired-end 150 bp sequencing (PE150) on an Illumina NovaSeq platform.

### 2.7 Bioinformatic analysis

#### 2.7.1 Preparation of snRNA-Seq count matrices

Raw fastq sequence files were quality controlled with FastQC (Simon Andrews et al., 2010). Data were then processed with the Parse Biosciences SplitPipe software (split-pipe v1.1.2) with mode ‘all’ for zebrafish and medaka separately (https://www.parsebiosciences.com/). Zebrafish data were aligned to the *Danio rerio* GRCz11 assembly (ensembl.org/Danio_rerio/Info/Index) and medaka data were aligned to the *Oryzias latipes* ASM223467v1 assembly (ensembl.org/Oryzias_latipes/Info/Index). The split-pipe software generates quality control and cell-calling data and expression matrices, gene features, and cell metadata files (all_genes.csv, cell_metadata.csv, and count_matrix.mtx) for further processing.

#### 2.7.2 Processing of count matrices

The output files from split-pipe, for each species separately, were imported into the R software environment (Team, R.C., 2021) with the ReadMtx function in the Seurat v5 package (Hao et al., 2024). Data were filtered (minimum features per cell = 100, minimum counts per cell = 400, minimum cell per gene = 50, maximum mitochondrial content = 10%) and processed through the standard Seurat processing pipeline: SCTransform; RunPCA; FindNeighbors; FindClusters; and RunUMAP.

#### 2.7.3 Cross-species analysis

To directly compare the expression levels of genes in zebrafish and medaka cells, ortholog tables were used to identify pairs of genes in the two species. The getBM function from the R package biomaRt (Durinck et al., 2009) was used to extract one-to-one orthologs (‘ortholog_one2one’) between the species from the Ensembl database. A common identifier was prepared for each gene in the ortholog pair: the zebrafish gene name if present and the medaka name otherwise. New gene features files were created with the common cross-species gene names and used to import (as above) the expression data for the two species separately. This removes some data from the analysis; however, genes that are not present in the genomes of both species are not informative in this analysis. After import, systematic, species-specific biases were removed by integrating the data. The RunHarmony function from the harmony package (Korsunsky et al., 2019) was used to integrate the data from the two species and generate a combined data object with integrated cell embeddings to use for further processing and analysis. Differential expression was performed between clusters and between cells from the species with the FindAllMarkers and FindMarkers (after running PrepSCTFindMarker) on the integrated dataset. The propeller function from the speckle package (Phipson et al., 2022) was used to examine cell numbers between clusters and species. Groups of differentially expressed genes were submitted to ShinyGO (Ge et al., 2020) to identify any enriched biological pathways. Gene expression of marker genes and differentially expressed genes was visualized with the FeaturePlot and VlnPlot functions of Seurat with the integrated dataset and cluster/UMAP embeddings.

#### 2.7.4 Quantitative PCR (qPCR) and cross-species normalization

RNA extraction was carried out using vertebrae from a distinct subset of six-month-old adult fish. Zebrafish were processed individually, but medaka were pooled as per Table 1. Total RNA was extracted using the Qiagen RNeasy® Mini Kit with on-column DNase I treatment, following QIAzol–chloroform phase separation according to the manufacturer's instructions. RNA quantity and purity were assessed with a NanoDrop spectrophotometer (Thermo Fisher), and RNA integrity was evaluated on an Agilent TapeStation 4200. cDNA was synthesized from 10 μL of purified RNA using random primers and Superscript II reverse transcriptase (Thermo Fisher Scientific). The resulting cDNA was diluted to 5 ng/μL in RNase-free water. qPCR reactions were performed in triplicate using SYBR Green Master Mix (Primer Design, UK) on a Stratagene Mx3000P Real-Time PCR System (Agilent Technologies) under standard cycling conditions: 95 °C for 2 min, followed by 40 cycles of 95 °C for 15 s and 60 °C for 1 min. Melt-curve analysis was conducted to confirm single amplicon specificity.

The target panel included six genes identified as differentially expressed in medaka relative to zebrafish from the snRNA-seq analysis, together with two osteoblast (*sp7* and *sparc*) and two osteocyte (*sost* and *fgf23)* marker genes. β-actin was used as a housekeeping gene in each species. For each biological sample, the cycle threshold (Ct) value of each target gene was normalised to the geometric mean of the Ct values of *sp7* and *sparc* from the same sample, yielding a ΔCt value relative to the bone markers.

For each biological sample, the ΔCt value was calculated as: ΔCt = Ct_(target)_ − Ct_(β-actin)_ − [(Ct_(sp7)_ + Ct_(sparc)_) / 2]. Relative expression values were calculated using the 2^ꟷ ΔCt^ (Livak and Schmittgen, 2001). An unpaired *t*-test was applied to compare gene expression between the two species. Primer sequences are listed in Supplementary Table 2.

### 2.8 Statistical analysis

All statistical analyses were performed using appropriate GraphPad Prism software. Gene expression data are presented as mean ± standard deviation (SD). An unpaired *t*-test was used to compare gene expression between the two species. A *p*-value of <0.05 was considered statistically significant.

## 3 Results

### 3.1 Expression of classic osteocyte genes is restricted to osteoblasts in medaka bone

H&E staining revealed the expected differences in vertebral cellularity between medaka and zebrafish. In zebrafish, vertebral bone sections displayed identifiable osteocytes within the mineralized matrix (yellow arrows, Fig. 1). Osteocytes were not uniformly distributed throughout the entire bone section and were found predominantly around the junction between the vertebral body and its processes in zebrafish, as previously reported (Ofer et al., 2021). In medaka, vertebral bone tissue lacked embedded osteocytes, and no osteocytes were observed elsewhere in the skeleton. In both species, sections showed well-defined spinal cord and notochord structures (S, NC, Fig. 1). Additionally, cells were observed lining both the inner and outer surfaces of vertebrae. Based on previous reports, these cells are likely to be matrix-forming osteoblasts (asterisk, Fig. 1) (Ekanayake and Hall, 1987; Ekanayake and Hall, 1988b).

**Fig. 1:**
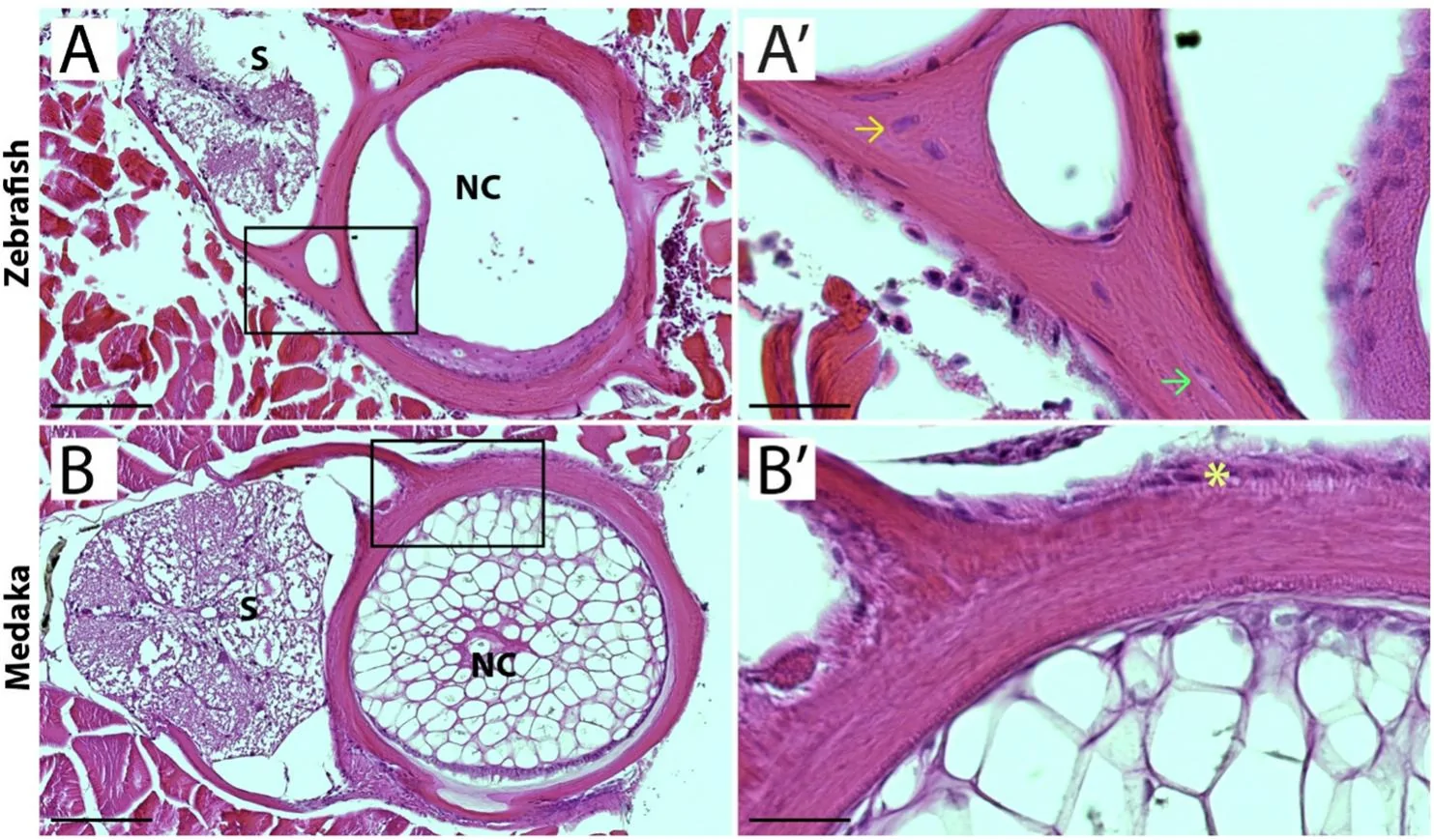
**Histological comparison of bone structure in medaka and zebrafish.** Representative H&*E*-stained sections of medaka and zebrafish vertebrae. Zebrafish vertebrae (A) exhibit osteocytes embedded within mineralized matrix (yellow arrow), with incomplete osteocyte within lacunae indicated by green arrows, and osteoblasts lining the bone (asterisk). Medaka bone exhibits an acellular structure lacking embedded osteocytes (B). In both species, the spinal cord (S) and notochord (NC) are clearly visible. Scale bars = 100 μm A, B, 25 μm A', B′.

Having confirmed the absence of osteocytes in Medaka, expression of classic osteocyte genes, sclerostin (*sost)* and fibroblast growth factor 23 (*fgf23)*, was analysed in zebrafish and medaka bone samples, via qPCR. Both genes were found to be expressed at low levels in zebrafish and medaka (Fig. 2A, D). Expression of both *sost* and *fgf23* was slightly higher in zebrafish (50% and 40% lower than osteoblast markers, respectively) compared to medaka (70% and 90% lower than osteoblast markers), although differences were not significant. (Fig. 2 A, D). These data imply that a non-osteocyte bone cell type, namely the osteoblast, may compensate for the expression of osteocyte-functional genes in medaka. To explore this possibility, spatial analysis of *sost* and *fgf23* gene expression was performed via RNA-FISH. *Sost* expression was identified in the osteocytes of zebrafish (Fig. 2 B) but was absent from osteoblasts. In medaka, *sost* expression was seen in osteoblasts (Fig. 2 C). Expression of *fgf23* in zebrafish was again confirmed in osteocytes, but also in osteoblasts lining the bone (Fig. 2 E), and in osteoblasts of medaka (Fig. 2 F). Notably, the expression of *sost* and *fgf23* was detected only in a subset of osteocytes within the zebrafish vertebral bone; this aligned with data in medaka bone, where *sost* and *fgf23* expression was not observed in all osteoblasts lining the bone surface. Confirmation that the bone lining cells in medaka and zebrafish were osteoblasts was achieved using RNA-FISH to identify localisation of collagen type I alpha 1a (*col1a1a*) expression at the cell layers on the vertebrae surface, and the presence or absence of cells was confirmed using DAPI (Fig. 2 G-J).

**Fig. 2:**
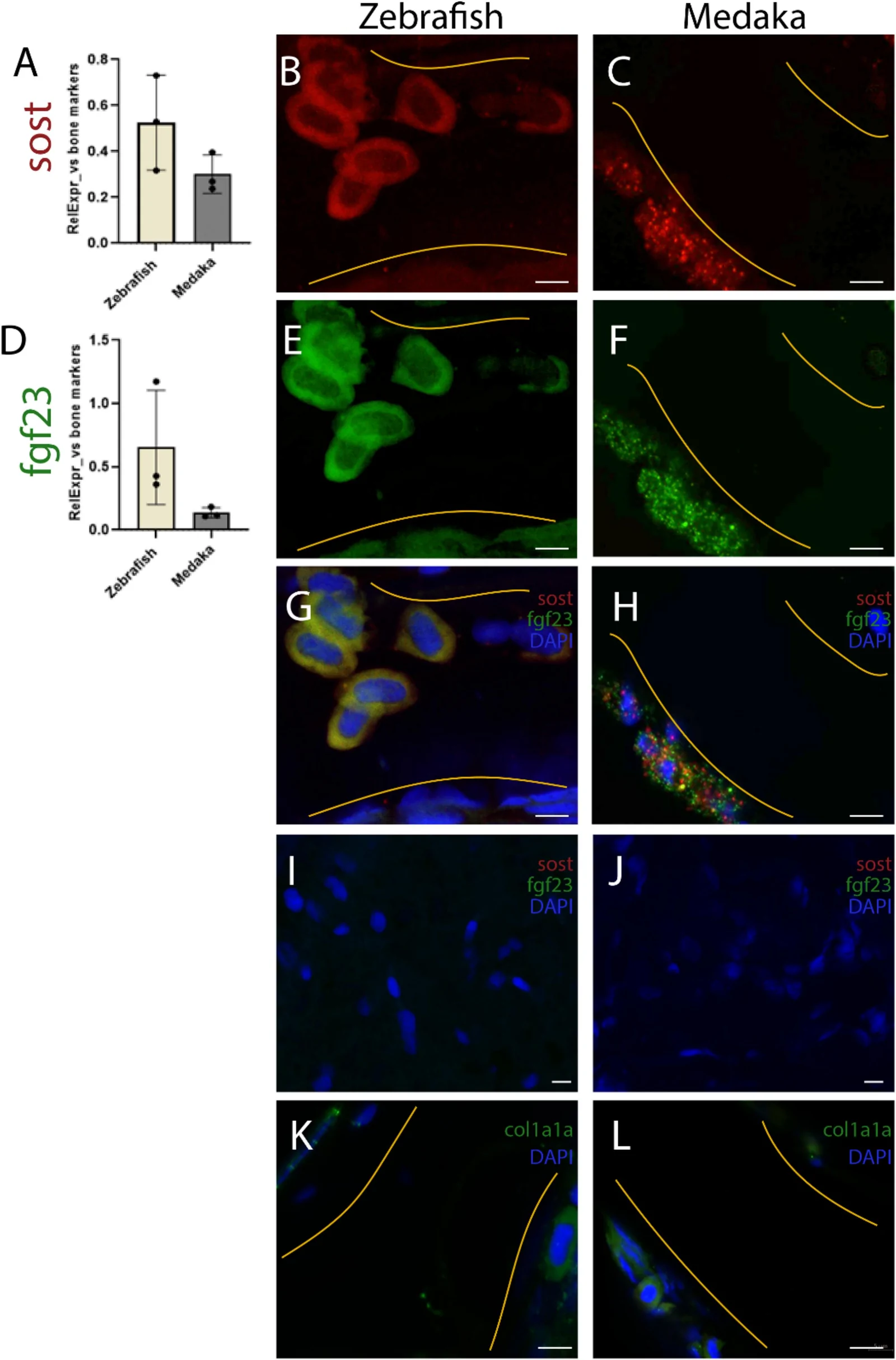
**Osteocyte markers are expressed in the osteoblasts of medaka bones.** Gene expression of the classic osteocyte markers, *sost* and *fgf23*, was measured using qPCR, normalised to osteoblast markers *sp7* and *sparc* (A, D) and localisation confirmed by RNA-FISH (B, C, E-L). *Sost* expression was detected at a mean of 50% lower than osteoblast markers in zebrafish and 70% lower in medaka; the difference in expression levels was not significant (*p* = 0.156 by Student's *t*-test). Spatial analysis confirms expression of *sost* in the osteocytes of zebrafish (B), and in osteoblasts of medaka (C). *Fgf23* expression was found to be 40% lower than osteoblast control genes in zebrafish, but 90% lower in medaka (D). This difference of expression was not significant (*p* = 0.119 by Student's *t*-test). Expression was observed in osteocytes and osteoblasts of zebrafish (E). Expression was observed in the osteoblasts of medaka (F) and absent in muscle of both zebrafish and medaka (I, J). Col1a1 staining identifies osteoblast-rich bone surfaces in zebrafish and medaka (*K*,*L*). Yellow lines delineate the periosteal and endosteal surfaces. Scale bars, 5 μm.

### 3.2 Cross-species integration of bone-derived nuclei resolve a cluster harbouring both an osteoblastic and ‘osteocyte’ marker gene signature

Having confirmed that medaka osteoblasts express classic osteocytic genes, snRNA-seq was employed to identify broad trends in expression of bone genes across distinct populations of bone cell nuclei isolated from these species. The zebrafish and medaka data were integrated using only the genes that were defined by Ensembl as one-to-one orthologs between the two species. Initial analysis identified 12 distinct transcriptional clusters in the zebrafish and medaka samples, each corresponding to a specific cell type based on marker gene expression; the number of clusters highlights the shared transcriptomes between species, and the cellular heterogeneity of our samples (Fig. 3A). Some clusters exhibited similar proportional representation between the 2 species (particularly clusters 2, 4, 6, 7, 8 and 9), although there were some notable exceptions (Fig. 3 B). The nuclei in clusters 1 and 10 were more abundant in zebrafish compared to medaka, while nuclei in clusters 0, 3, 5 and 11 were more plentiful in medaka than zebrafish. The clusters this analysis identified were found to exhibit gene markers suggestive of a range of cell types, including chondrocytes, germ cells, neural cells, blood cells, muscle cells, and mesenchymal cells. The presence of these clusters provides clear evidence for the inclusion of non-skeletal cell populations, likely originating from contaminating soft tissues, which are technically difficult to remove fully from these fish samples. Nevertheless, analysis of genes enriched in cluster 2 indicated a clear enrichment for genes associated with osteoblast and osteocyte function compared with other clusters, and that this enrichment was evident in nuclei from both species. It is notable, however, that overall expression levels of osteoblast- and osteocyte- related genes in cluster 2 are low, and that neural-like cells are also present in this cluster.

**Fig. 3:**
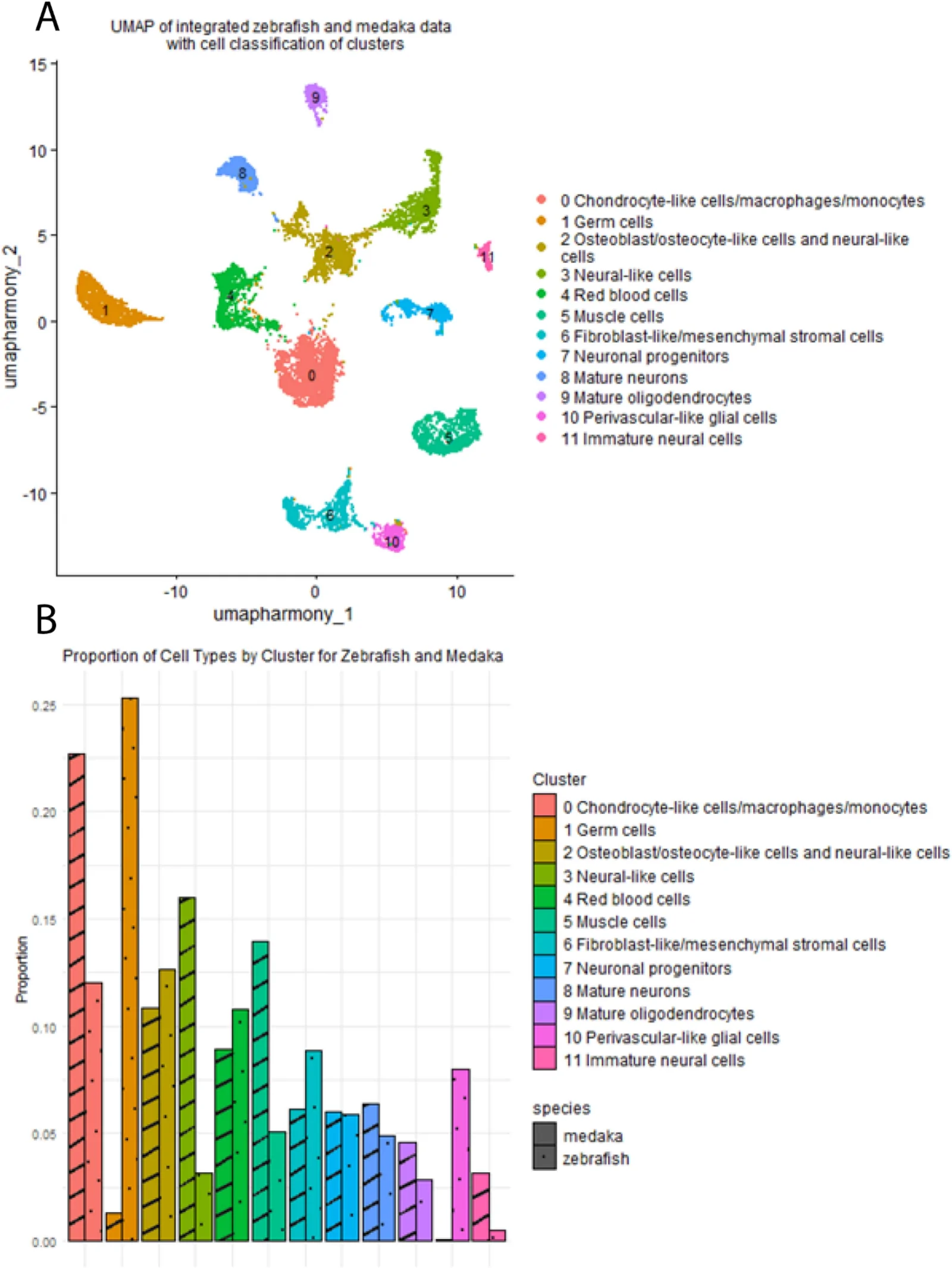
**Harmony-based integration and clustering of medaka and zebrafish snRNA-seq datasets.** Harmony-based integration of single-nucleus RNA-seq data from medaka and zebrafish identified 12 transcriptionally distinct clusters annotated by cell type. (A) UMAP visualisation showing integrated clustering of nuclei from both species. (B) Proportional distribution of clusters across medaka and zebrafish.

Further analysis of cluster 2 identified the expression patterns of recognized osteoblast genes, revealing many to be expressed in both zebrafish and medaka, albeit at different levels and proportion of nuclei, in particular, *col1a1a, col1a1b* (the zebrafish and medaka ortholog of the mouse osteoblast marker gene *Col1a1)*, osterix (*sp7)*, osteonectin (*sparc)*, bone gamma-carboxyglutamate protein-like *(bglapl;* zebrafish have a functional ortholog of the mouse *Bglap* osteoblast marker gene*,* but medaka do not) and osteopontin (*spp1*) are all expressed (Fig. 4). A key marker of mineralization in skeletal tissue, tissue-nonspecific alkaline phosphatase (*alpl*) is seen at low levels in cluster 2, but greater expression is noted in cluster 6 (fibroblast/MSC cells) for both medaka and zebrafish. The expression of the ortholog of the mammalian Runt-related transcription factor 2 (*Runx2)* gene (*runx2b*) was moderate in cluster 2 with lower expression in cluster 3 (Fig. 4). Collagen type 1 is the major structural protein of bone and two (*col1a1a* and *col1a1b)* of the three type 1 collagen genes found in zebrafish (Gistelinck et al., 2016), were expressed by ∼50% of nuclei whereas the other osteoblast genes were expressed by less than 50% of nuclei in cluster 2. Not all osteoblast genes were expressed equally in cluster 2 by nuclei isolated from medaka and zebrafish. The expression of *col1a1a*, *col1a1b*, *runx2b* and *sparc* was higher in medaka, whereas *sp7* and *spp1* showed higher expression in zebrafish. In all, this analysis confirmed that osteoblast genes are expressed in cluster 2 at higher levels than in other clusters; it must be noted that expression levels are low and restricted to relatively few nuclei within the cluster. The piezo1 gene, involved in mechanotransduction in mammals, is expressed but at a low level in both species, including in cluster 2 (Fig. 4). piezo2 (piezo2b in zebrafish) is not expressed above the level of detection in either species.

**Fig. 4:**
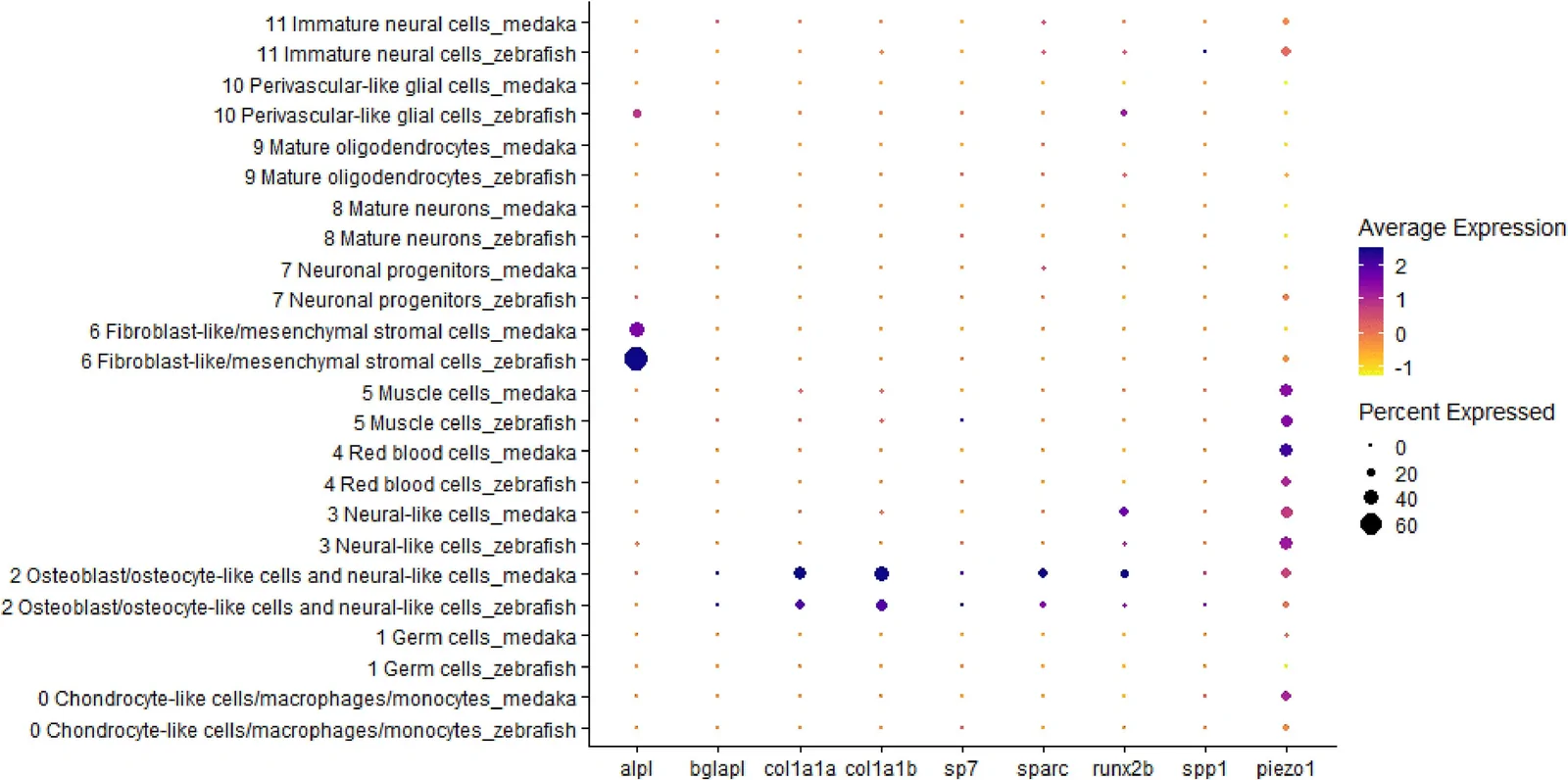
**Expression of osteoblast-related genes in single nuclei from bone tissue of medaka and zebrafish.** Dot-plots showing the expression of osteoblast-related genes across identified clusters and in both species. The size of each dot represents the proportion of nuclei in a cluster expressing the gene.

The key hypothesis of this study was to identify potential cell types that may act in a compensatory manner to overcome the lack of osteocytes in medaka. We therefore assessed the expression patterns of 26 genes previously shown to be highly enriched in murine osteocytes in medaka and zebrafish (Youlten et al., 2021). In addition to these 26 genes, fgf23 and pth1r (parathyroid hormone receptor 1) were also assessed. Of these 28 genes, 16 orthologues were identified in our dataset in either zebrafish or medaka.

Analysis revealed that *sost*, *emilin2a*, *dkk3b*, *suchi domain containing 5* (*susd5*), and *fibulin-7* (*fbln7*) were expressed at low levels in cluster 2 and by a small proportion of nuclei in both species (Fig. 5). Each of these genes was more strongly expressed in other clusters. *Emilin2a* and *dkk3b* were predominantly expressed in cluster 11 (immature neural cells), *iroquois homeobox 5* (*irx5*) homolog (*irx5a*) is expressed more highly in cluster 3, neural-like cells, *neuronal guanine nucleotide exchange factor* (*ngef*) and *cilia and flagella associated protein 70* (*cfap70*) (clusters 6 and 7, fibroblast/MSC and neuronal progenitors), *proline rich 15 like b* (*prr15lb* - clusters 6, 7 and 10, perivascular-like glial cells) and *semaphorin-3E* (*sema3e*, clusters 5 and 8, muscle and mature neurons). In cluster 2, the expression of *irx5a*, *dkk3b*, *emilin2a*, *cfap70* and *susd5* was higher in medaka, while *fbln7*, *prr15lb*, *sost*, *seema3a* and *ngef* showed higher expression in zebrafish. Whilst *fgf23* was not identified by Youlten, its expression by a small number of nuclei was higher in zebrafish bone and localized to cluster 2, confirming our expression data. Among the remaining genes, Wnt family member 1 (*wnt1*) and notum palmitoleoyl-protein carboxylesterase homologs (*notum 1* and *notum 2*) were detected but exhibited very low expression levels in both species. The pth1r gene is expressed at low levels in zebrafish and very low levels in medaka. The absent genes include matrix extracellular phosphoglycoprotein (*mepe*), fibroblast growth factor 9 (*fgf9*), dickkopf (*dkk1*), sosondowah ankyrin repeat domain family member B (*sowahb*), transforming growth factor beta 2 (*tgfb2*), *A930001C03Rik, Obcdi053500*, C2H2-type zinc finger transcription factor (*zic3*), acid-sensing ion channel subunit 3 (*asic3*), iroquois homeobox 6 (*irx6*), and dentin matrix acidic phosphoprotein 1 (*dmp1*).

**Fig. 5:**
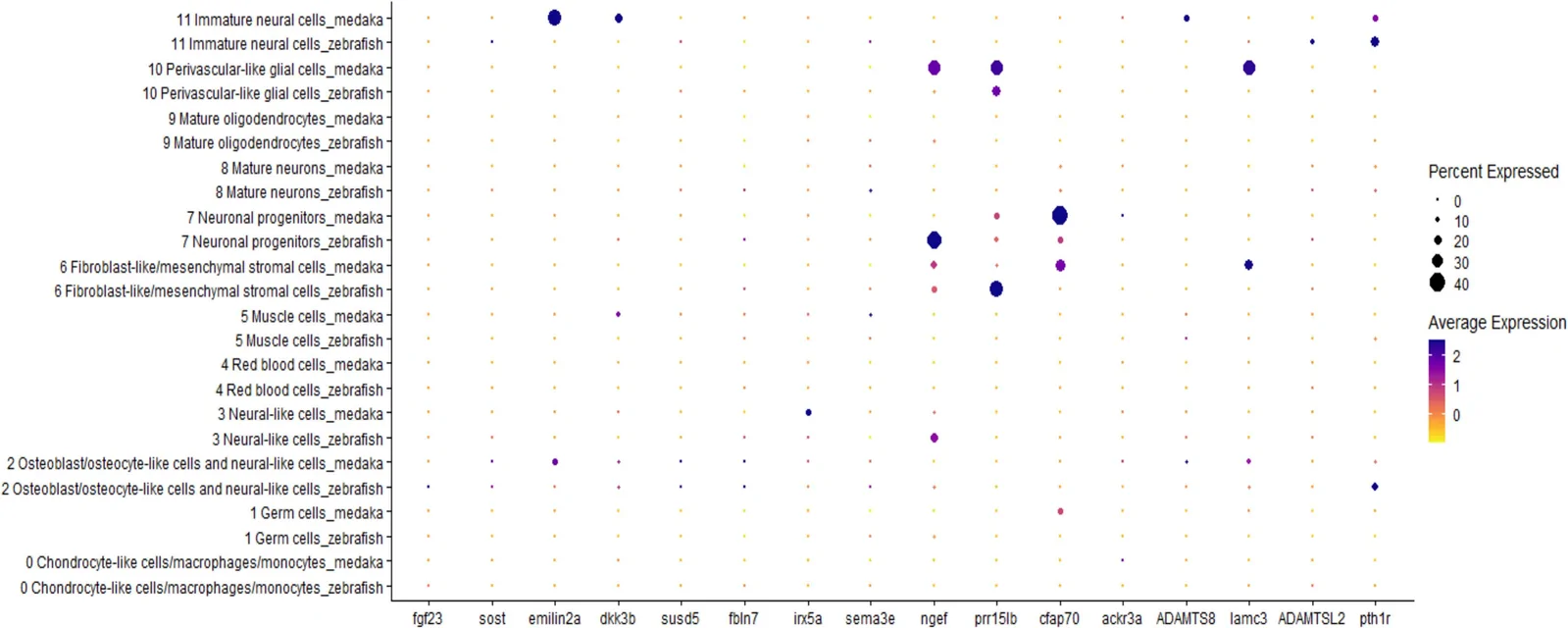
**Expression of osteocyte-related genes in single nuclei from bone tissue of medaka and zebrafish.** Dot-plots showing the expression of osteocyte-related genes across identified clusters and in both species. The size of each dot represents the proportion of nuclei in a cluster expressing the gene.

### 3.3 Upregulation of stimuli-sensing pathways in medaka vertebrae

To further understand the pathways involved in anosteocytic mechano-adaptation and medaka bone (re)modelling, pathway enrichment analysis by ShinyGO was employed, identifying enriched pathways across the full dataset of highly differentially expressed genes (*n* = 306) in medaka compared to zebrafish bone (Table 2). The stimuli-sensing channels pathway (R-DRE-2672351) contains cellular sensors for various physical, chemical, pressure and thermal stimuli. Of the 76 pathway genes, six were enriched - *clcnk, trpc5a, ano9b, trpm2,* and *trpm1b* (fold enrichment = 7.91; FDR = 0.0234). ShinyGO also listed *trp5b* as part of this pathway, likely due to its paralogy with *trp5a*, but *trp5b* was not detected in our data set. The transient receptor potential (TRP) ion channel pathway (R-DRE-3295583) was significantly enriched in 4 of the 25 genes that comprise this pathway (fold enrichment = 16.03, FDR = 0.0234). The dataset included the genes *trpc5a, trpm2, and trpm1b*, which were also enriched in the stimuli-sensing channels pathway. As the TRP pathway is a member of the stimuli-sensing channels pathway, this was as expected. These findings suggest that, relative to zebrafish, medaka bone is characterized by an enrichment of genes associated with stimuli sensing, despite the absence of osteocytes.

**Table 2:** Pathway enrichment analysis of genes highly expressed in medaka bone tissue compared to zebrafish bone tissue.

Enrichment FDR	nGenes	Pathway genes	Fold enrichment	Pathways (click for details)
2.3E-02	4	25	16	R-DRE-3295583 TRP channels
2.3E-02	6	76	7.9	R-DRE-2672351 Stimuli-sensing channels

Pathway-enriched channel genes exhibit distinct expression patterns across the clusters in both medaka and zebrafish (Fig. 6). In both fish, *trpc5a* (clusters 6, 8 and 10), *clcnk* (cluster 7), and *trpm1b* (cluster 11) showed stronger expression in neural and fibroblast-like clusters rather than the osteoblast/osteocyte cluster (cluster 2). In contrast, *ano9b* and *trpm2* were poorly expressed in all clusters. Of the genes analysed in the stimuli-sensing pathway, *trpc5a* exhibited the strongest expression in the most significant number of nuclei present in cluster 2, but caution should be taken in the interpretation of these findings, as less than 25% of nuclei in this cluster express *trpc5a*, and average expression is below 1. The slight increase in expression of *trpc5a* in cluster 2 of medaka was supported by RT-qPCR analysis of medaka vertebrae, in which *trpc5a* was more highly expressed than in zebrafish vertebrae. The remaining pathway-enriched channel genes showed a trend towards higher expression in medaka, but these differences did not reach statistical significance (Fig. 7A). Spatial expression analysis by RNA-FISH for the *trpc5a* gene showed low expression in osteocytes, but similar, higher, expression in a subset of osteoblasts in both zebrafish and medaka, with the higher percentage of osteoblasts within the bone of medaka, perhaps explaining the increase seen in snRNA-seq (Fig. 7 B).

**Fig. 6:**
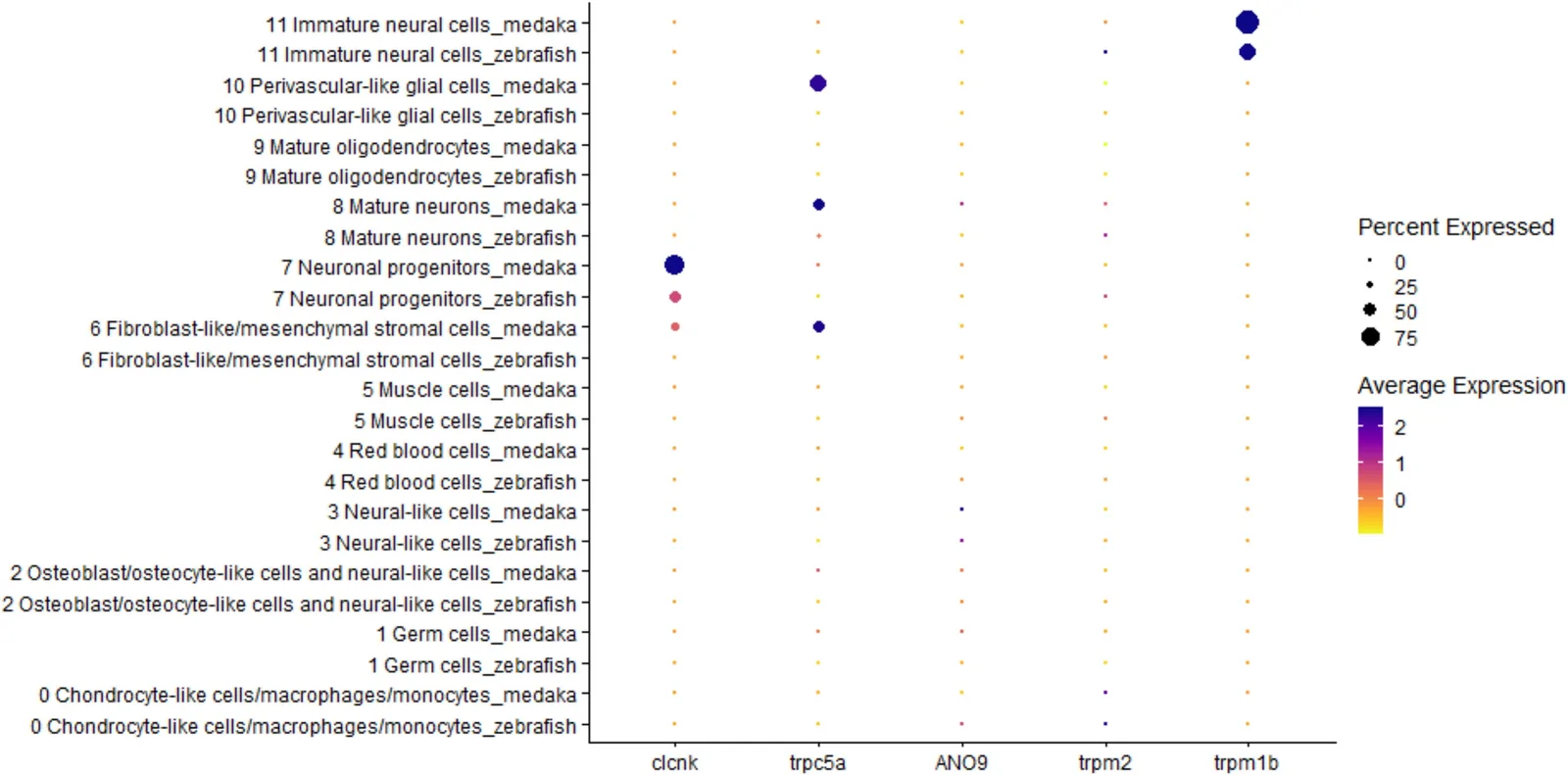
**Expression of enriched pathway genes in single nuclei from bone tissue of medaka and zebrafish.** Dot-plots showing the expression of osteocyte-related genes across identified clusters and in both species. The size of each dot represents the proportion of nuclei in a cluster expressing the gene.

**Fig. 7:**
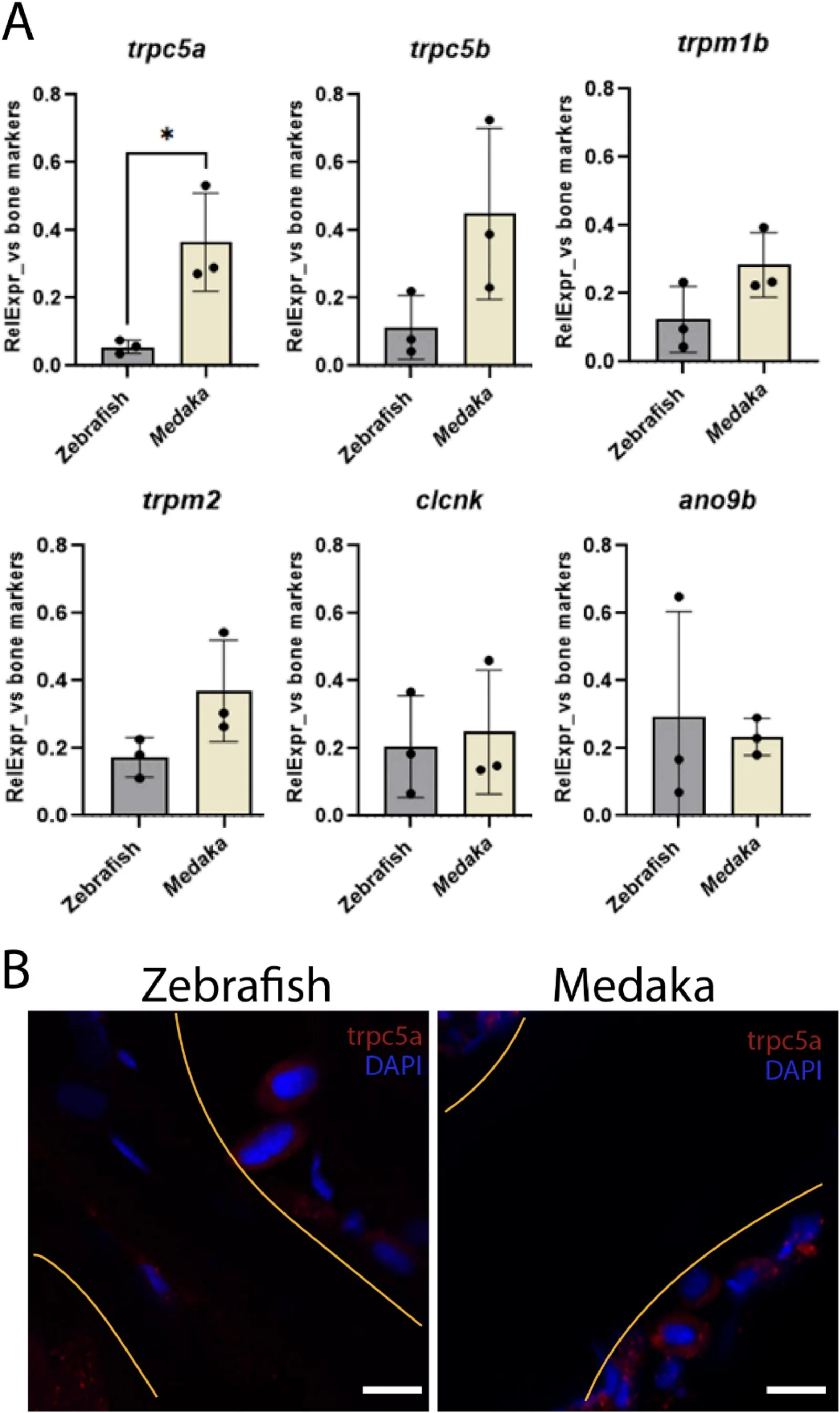
**Expression analysis of pathway-enriched TRP channel genes in zebrafish and medaka bone.** Relative expression of pathway-enriched channel genes (*clcnk, trpc5a, ano9b, trpm2, trpm1b, trpc5b*) by qPCR, normalised to the osteoblast markers *sp7* and *sparc* (A). All genes other than *ano9b* were expressed at higher levels in medaka than in zebrafish. *trpc5a* expression was significantly enriched in medaka (*p* < 0.05 by Student's *t*-test, bars represent mean ± SEM). Expression of *trpc5a* was analysed spatially using RNA-FISH (B); expression was restricted to a subset of osteoblasts in both zebrafish and medaka. Scale bars = 5 μm. Yellow lines denote periosteal and endosteal surfaces.

## 4 Discussion

Osteocytes, the most abundant (>90%) and longest-lived cell in the adult skeleton, are fundamental to the structure and function of bone (Dallas et al., 2013). They are considered terminally differentiated, osteoblast-derived cells resident in mineralized bone, and their dendritic morphology allows them to retain direct contact with bone-forming osteoblasts to create multicellular networks permeating the bone matrix (Holmbeck et al., 2005). Once considered passive ‘place-holders’, osteocytes have, over the last 30 years or so, been emerging as versatile orchestrators of bone (re)modelling (Dallas et al., 2013). It is now established that osteocytes produce pro-osteogenic factors (Pitsillides et al., 1995a; Rawlinson et al., 1996) and that their function underpins the increased bone mass in response to mechanical loading. This was confirmed in mice, where osteocytes were ablated by directing the diphtheria toxin receptor to osteocytes using the DMP1 promoter (Tatsumi et al., 2007; Al-Jazzar et al., 2016).

Osteocytic bone forms as osteoblasts become embedded in the mineralizing matrix. Once embedded, osteocytes put out cytoplasmic processes that establish intercellular communications via a lacunar-canalicular network that coordinates strain sensing and remodelling of bone. Considering this primacy of osteocytes in bone biology, the existence of modern fish whose bones are structurally and functionally normal despite lacking osteocytes is developmentally and functionally intriguing. How anosteocytic bone forms is unclear, but the prevailing views are that there is polarized secretion of osteoid towards the existing bone surface, thus avoiding osteocyte entrapment, or alternatively, normally formed osteocytes die via apoptosis followed by mineralization of the lacunar space (Weiss and Watabe, 1979; Moss, 1961a; Moss, 1961b; Ofer et al., 2019b). Functionally, an adaptive modelling response to physical stimuli such as strenuous swimming has been widely reported in anosteocytic fish (Tauer et al., 2024; Atkins et al., 2015a; Kranenbarg et al., 2005a; Kranenbarg et al., 2005b). Freshwater fish such as cichlids also show pharyngeal jawbone reshaping and internal trabecular thickening upon feeding-related mechanical stimulation (Huysseune et al., 1994; Muschick et al., 2011). Direct mechano-adaptive links have been afforded in anosteocytic fish by controlled application of load (magnitude/direction/duration) to the tilapia operculum (bony gill cover), which adapted morphologically and became stiffer (Atkins et al., 2015b). Furthermore, heavily loaded anosteocytic bone in the large, pointed rostra of billfish (e.g.*,* swordfish ‘bills’) contains a dense population of secondary osteons; structures considered definitive evidence for (re)modelling in vertebrates (Atkins et al., 2014). As in mammals, morphological evidence of (re)modelling in the blue marlin suggests correlations with ontogeny and mechanical demands.

The knowledge that anosteocytic bone can sense and respond to the need for architectural adaptation and tissue replacement and regulate bone (re)modelling processes without osteocytes, indicates that alternative mechano- and homeostasis-sensing mechanisms are present in bone of anosteocytic fish. Alternative mechanisms have previously been reported, in which vertebrae of anosteocytic medaka and other (Adrianichthyinae) ricefish reveal an altered expression of many genes, including *sost*, *col2α1α* and *myoc,* in response to swim training (Ofer et al., 2019a; Tauer et al., 2024). This valuable insight was extended in the present study, where we sought to identify the cell type(s) expressing recognized osteocyte genes under basal, non-loaded conditions. Such an approach has allowed us to examine whether anosteocytic fish have evolved to express osteocyte genes in non-osteocyte cells and/or if alternative mechano-sensing molecular cues have become more prominent. The ability to replace the repertoire of osteocyte functions by another cell type would be a significant observation as anosteocytic bone of neoteleosts does not resemble mammalian bone that contains osteocytes that are necrotic or dead (Tatsumi et al., 2007). Reduced osteocyte viability is associated with accelerated bone resorption and elevated *Tnfsf11* (RANKL) expression and decreased expression of *Fgf23*, *Sost*, *Mepe*, *Phex* and *Pdpn* (Tatsumi et al., 2007; Komori, 2013). These genes are vital for bone homeostasis and their aberrant expression results in mice with fragile cortical bone with increased porosity and micro-fractures, osteoblastic dysfunction, and trabecular bone loss with microstructural deterioration (Tatsumi et al., 2007).

Our first approach was to determine which cells in medaka and zebrafish bone expressed *fgf23* and *sost,* recognized markers of the mammalian osteocyte. RNA-FISH localized transcripts of both genes to osteoblasts on the medaka bone surface, whereas in zebrafish bone, *sost* and *fgf23* expression was preferentially localized to osteocytes rather than osteoblasts. This provided initial evidence that in medaka, the function of the osteocyte is transferred to the osteoblast, and therefore, we next completed an snRNA-seq study to provide further confirmatory data.

Analysis of the snRNA-seq data, together with recognition of marker gene expression, identified 12 distinct cell-type clusters in both the medaka and zebrafish datasets. Many of these clusters, however, contained non-skeletal cell populations, bringing into question their relevance to this study. We nevertheless included them in some downstream data analysis, but future studies should optimize vertebral bone dissections to minimise the contamination by soft tissue. This is particularly relevant in studies of bone, where the cellularity of the tissue is so much lower than that of the surrounding soft tissue, such as muscle. Despite these limitations, cluster 2 from both species contained archetypal osteoblast genes such as *col1a1a, col1a1b*, *sp7*, *sparc*, *bglapl*, *runx2b*, *alpl* and *spp1*, congruent with the presence of osteoblasts in the bone of zebrafish and medaka (Ekanayake and Hall, 1988a; Weigele and Franz-Odendaal, 2016). The vertebral bone of both medaka and zebrafish is lined with an inner and outer layer of osteoblasts, but in medaka, only the osteoblasts on the outer surface are responsible for periosteal osteogenesis (Ekanayake and Hall, 1988a; Ekanayake and Hall, 1987). Cluster 2 also contained genes considered to be part of the osteocyte transcriptome signature. Among the direct orthologs (defined by Ensembl) or family members where no orthologs exist of 26 genes identified by Youlten and colleagues (Youlten et al., 2021) as most enriched in murine osteocytes, we found *sost*, *emilin2a*, *dkk3b*, *susd5*, and *fbln7* to be expressed in the vertebral bone of both fish species. It is noteworthy that in cluster 2 of both fish species these genes were expressed at low levels and by a small proportion of nuclei, which for *sost* expression was surprising, knowing its specificity to the osteocyte and our zebrafish RNA-FISH data (Fig. 2). Moreover, *emilin2a* and *dkk3b* were most predominantly expressed in cluster 11 of medaka, which might provide additional evidence for anosteocytic fish expressing osteocyte genes in non-osteocyte cells. In contrast to *emilin2a*, *susd5*, and *fbln7,* which have no reported role in the skeleton, the *sost* and *dkk3* gene products are potent negative modulators of osteogenesis (Balemans et al., 2001; Aslan et al., 2006). The similarly low expression of *sost* and *dkk3b* in cluster 2 by zebrafish and medaka bone is initially surprising, given the absence of osteocytes in medaka bone. However, this may be explained by the RNA-ISH data showing that *sost* is more highly expressed by medaka osteoblasts compared to zebrafish osteoblasts. Similarly, *fgf23* is a recognized mature osteocyte-derived phosphaturic hormone and its expression in medaka bone, albeit at lower levels than in zebrafish bone, is evidence of a non-osteocytic source, which is consolidated with the osteoblast localisation of *fgf23* in medaka bone (Ubaidus et al., 2009).

The expression of recognized osteocyte genes by osteoblasts in medaka bone strongly suggests that in anosteocytic bone, the osteocyte's role is assumed by osteoblasts that may have accessed osteocyte potential earlier in the osteogenic lineage. Mammalian osteoblasts express low levels of *Sost* (Winkler et al., 2003; Collette et al., 2013) but are responsive to changes in their strain environment (Dodds et al., 1993; Lu et al., 2012; Pitsillides et al., 1995b). Nevertheless, osteocytes, unlike osteoblasts, are perfectly located and interconnected within bone's 3-dimensional structure to measure strain and transfer this information to bone surfaces where changes in architecture and mass could be realised. If, on the other hand, osteoblasts are the main strain sensors in medaka, then the need for measuring mechanical strain within the bone becomes redundant. This implies that mechano-adaption can occur with strain being detected solely by osteoblasts on the bone surface in anosteocytic bone.

Similar observations were made in a previous study of medaka bone, where *sost* expression was localized to chondrocytes, chordoblasts and also osteoblasts on the surfaces of the vertebral bone and neural spine (Ofer et al., 2019a). These authors also reported that *sost* expression decreased and *Col1a1* expression increased in medaka bone in response to resistance swimming, and furthermore that morpholino knockdown of *sost* expression in medaka resulted in increased bone formation (Ofer et al., 2019a). The reduction in osteocyte-derived *Sost* expression in mediating load-induced bone formation is also observed in mammals (Bellido, 2006) and suggests that medaka (and possibly other neoteleosts) have evolved a non-osteocytic mechanism to utilize sclerostin to control osteoblast function and bone formation. However, the expression of *sost* by zebrafish osteocytes in this study is in contrast to the Ofer study (Ofer et al., 2019a), where the expression of *sost* in zebrafish was predominantly found in chordoblasts and osteoblasts and not osteocytes, which expressed *sost* very weakly, if at all (Ofer et al., 2019a).

Understanding how osteoblasts detect changes in strain could provide insights into the evolution and diversity of skeletal biology across species. From the literature, we know that in mammalian systems, bone cells respond directly to mechanical deformation and fluid shear stress through multiple mechanosensory pathways, including mechanosensitive ion channels *(*e.g. PIEZO and TRP channels), integrins, and cell-to-cell contacts (Wang et al., 2022; Kaneko et al., 2014)**.**. TRP channels are predominantly localized to the plasma membrane and often within that of the primary cilium (Verbruggen et al., 2023). They are evolutionarily conserved cellular sensors that transduce and integrate environmental signals. They have broad selectivity towards cations such as sodium, magnesium and calcium that enter cells in response to a variety of physiological stimuli, which include, among others, temperature, light, pain and mechanical forces (Zhang et al., 2023). Of the known 28 different mammalian TRP channel proteins, there are seven subfamilies - TRPA, TRPC, TRPM, TRPML, TRPN, TRPP and TRPV based on sequence homology (Huang et al., 2024). From the differentially expressed genes found in this study, both the stimuli-sensing channels pathway and the TRP ion channel pathway were found to be enriched. Of the genes that comprise these pathways *trpc5a, trpm2* and *trpm1b* were enriched as were the chloride channels *ano9b* and *clcnk*. Although the snRNA-seq data revealed that *trpc5a* was poorly expressed in cluster 2, we did, by RNA-FISH, show that trpc5a was expressed by osteoblasts in medaka and both osteoblast and osteocytes in zebrafish. TRP family members are widely distributed throughout many cell types, including those of the skeleton, where they have been associated with regulating gene expression in osteoblasts, osteocytes and osteoclasts (Abed et al., 2009; Cao et al., 2019; Liu et al., 2022; Lee et al., 2015; Lieben and Carmeliet, 2012). While most TRP subfamily members show mechano-sensing characteristics, their precise role is unclear, and they may act as modulators of the response rather than as primary mechano-transducers (Jin et al., 2020; Christensen and Corey, 2007; Perin et al., 2023). Nevertheless, in response to hypo-osmotic stress, TRPM3 and TRPV4 channels facilitate increased intracellular calcium levels, which in turn regulate RANKL and NFATc1 expression in mouse osteoblasts (Son et al., 2018). Also, TRPV4 localized to the cilia of osteocytes can trigger a calcium influx when subjected to fluid flow stress (Lee et al., 2015; Moore et al., 2018; Gu et al., 2026).

Recent evidence indicates that zebrafish attain peak bone mass between 2 and 4 months of age (Gelles et al., 2025) and therefore six-month-old zebrafish and medaka, as used in this study, represent skeletally mature adults. We selected this age because the vertebral skeleton is fully developed and maintained, allowing comparison of osteocytic and anosteocytic bone under mature physiological conditions while minimizing variability associated with ongoing skeletal growth. Consequently, our findings primarily reflect mechanisms operating in mature bone rather than during peak skeletal acquisition. Whether the relative contributions of osteoblasts and other skeletal cell populations to mechanoadaptation differ during the earlier stages of skeletal development remains unknown. Future studies incorporating younger fish and controlled mechanical loading paradigms will be important to determine how alternative mechanosensory mechanisms are established during skeletal maturation and whether they change across the lifespan.

In summary, the results of this study indicate that osteocyte marker genes are expressed in medaka bone despite its lack of osteocytes. Rather than the selective expression of *sost* and *fgf23* by osteocytes in zebrafish they are expressed by osteoblasts in medaka, suggesting that in medaka the function of the osteocyte is transferred to the osteoblast. While further work is required, deciphering the cells and factors underpinning anosteocytic bone's mechano-adaptive capacity has the potential to discover untapped potential for cell targets in bone therapeutics.

## CRediT authorship contribution statement

**S.N. Jayash:** Writing – review & editing, Writing – original draft, Methodology, Investigation, Formal analysis, Conceptualization. **D.R. Dunbar:** Writing – review & editing, Writing – original draft, Formal analysis. **S. Guizard:** Writing – review & editing, Formal analysis. **J. Furniss:** Writing – review & editing, Formal analysis. **R. Ruiz Daniels:** Writing – review & editing, Conceptualization. **A.A. Pitsillides:** Writing – review & editing, Conceptualization. **K.A. Staines:** Writing – review & editing, Conceptualization. **D. Robledo:** Writing – review & editing, Conceptualization. **C. Farquharson:** Writing – review & editing, Writing – original draft, Formal analysis, Conceptualization. **L.A. Stephen:** Writing – review & editing, Writing – original draft, Formal analysis, Conceptualization.

## Open access

For the purpose of open access, the authors have applied a CC-BY public copyright license to any Author Accepted Manuscript version arising from this submission.

## Funding

The authors are grateful to the 10.13039/501100000268Biotechnology and Biological Sciences Research Council (BBSRC) for supporting LAS via a Discovery Fellowship (BB/X009904/1) and for Institute Strategic Programme Grant Funding (BBS/E/RL/230001A and BBS/E/RL/230001C) to SNJ, JF, LAS, RRD, DR and CF. DRD and SG were funded by BBSRC Core Capability Grant BB/CCG2270/1 awarded to the Roslin Institute. We also acknowledge the 10.13039/501100000265Medical Research Council for funding to KAS (MR/V033506/1 and MR/R022240/2).

## Declaration of competing interest

None.

## Data Availability

The raw and sequencing data are available in the ArrayExpress database (www.ebi.ac.uk/arrayexpress) under accession number E-MTAB-16880, which is brokered to the European Nucleotide Archive (ENA).
