# Supplementary material for: Maintenance of bone health by osteoblasts in the absence of osteocytes: comparative analysis of gene expression in medaka and zebrafish vertebrae
Source: Bone Rep. 2026 Sep 30;31:101953. doi: 10.1016/j.bonr.2026.101953 (PMC13672807; doi:10.1016/j.bonr.2026.101953)
Supplement: Supplementary file 1 [file mmc1.docx]

**Supplementary Table 1.** Probe sequences used for in situ hybridisation (ISH). Probes were synthesised by Molecular Instruments.

| Species | Genes | Accession numbers | Sequence |
| --- | --- | --- | --- |
| Zebrafish | *sost* | XM_001340647.7 | UgCAAAACCUgUggUAgUUUAAAAAggCAAgCCUCUCCCgAAgAgAAgAAAACUggUCgAAgUUUgCCCgCUgAACggCUCgUUCUCggUUgUAUUAUgCAggUgUCUCUggCgCUCgUgCACUgCggCgCgCggCUgUUgCUgCUgCUgCUgCUUCAgggAUgUUUCACAgCUgCgCACggAUggAggCCUUUAAAAAACgAUgCUACAgAAAUUAUACCAgAAUACgCggAggACACgCACAAACCCgAggAgCAgAUACAACUgUCUgACAAUAACACAAUgAAUCgggCgAAgAACggUggAAggCggUCgUCAgCAAACACACggACUUAUggAgCCUCAgAACUCAgCUgUCgggAgUUgCgUUCgACCCgAUAUgUgACCgAUggAUCUUgCCgCAgUgUCAAACCCgUgAAggAACUUgUgUgUUCUggUCAgUgCCUUCCAgCUCACCUgAUgCCAAACUCAAUUCUACgggggAAAUggUggAgAAgCAgCUCCUCCgAUUACCgUUgCAUCCCUgCUCACUCCCgCACCCAgCggAUACAgCUCCAgUgUCCAAACggACCAAACCggACAUACAAAAUACgAgUUgUCACAUCUUgCAAgUgCAAACACUACACUCgACAACACAACCAAUCggAUgUCAAAgAgUCgUCCAAgAAACCACggCgCAAUAAgAAgCACCgCAgCAAAgUgCCgUCCAUCAACAgCAAUUCAUACUgAggggAAAAgAUgCAgAACUgAUACAgAAAACACUCUCUUgCACACAUACAAAAAAAAAgAAAgUACACCAAUACAUAUgCACACAUAUUACAgAAACUgCAAUCCUCAUCUACAAgggAAggAAUAAACgCAUUAgCAUUUACCAAgCUUgUUggCAUgCUAACAAgUUUCCAAUgUAUUUCAgAUgUCUCgCUgUUUgUAUgUgCUCUUgAgCCUAAAUCgUCCCAgUUUgAUCACAAUAUgCCAgCggACUgAgUgAAAAgACgUCCgCUgUUAUgCgAgAUUAgAUCAggCAAUgUgAUUUUUUUgAgggAggAgAAAUAUUCCUgCUCAAgCggUUCAUUUCAAgUUCACCgAAgUUCAUUgAAAggCUAACUUACgggCAAgCAUACUgAgUgCUCCACCAgUCCAgCAAgUgCUCAUgUgAAUgUUUgUgCACCAgAUUUgUCCUCUUUUgggAUUCggAgCAgUgUAUgAUUUgAgCgCUCAgCCgCgUCUggCCCAAAAgACACgCUgCUgCUgACAgACCACUgAAgCCCAgAAUUCUUAACCUUggUCACUUUUACCAggUCCUUUUUgACAggCUggggAgUCCAUgUgAgUgUCUgCAAAUUCACAUUUCCACAAgAAACUgAUCgACACUggCAgCUAgAgCCgCCUUACggAgAgUUCggAAAgUUgUgUCAgCCCUCCACggUCCAAgUCCCUggAggUCgAgAACAgCAAACggUAUUCCUgUgACUUgUCCUAAUUACCAAAUAgUUCCAUUCCUgUgUUUAggAUUUgAgUCAUAAAAAgCCAUUUCCUCUAgACAUggUCAgUUCCUgUUUgUUCgAACACCCgAAAAAgAggCCCACAUgUgUgCACgAAUCUgCAgCAAUUCCUCgCgggCAAAUAACCUUUACgCACCCUCCUUCAAUggAAACCAUUUggAgAUAUUCAgCUAUCUgCAAUCgCUAgCAggUUACCUACAUCCAUUACUgAAgUCUUCAgCAgAgAUgUCAUCCUUCCUgUCUCCACACAUgACCggAAAAgAACAAACAAAUAAgUgAACCAAUCUCUgAAAUCgUgggCUggAUgUUgAUUCUACACAggAUACAgAgAUCAgAgAAggCCAAUUgUUCAACAUUUUUUCUgCCCACCUCgAUgAUAUUUgCUCAUUUUUUCAAUUgUgUgUUUgAAAUAgCAACUggAAgggAUUUCUUAUAAUgUAAgCAgACUUgUCgUgAAUAgUAACUUgUACAUAgUAUACCUgUgUggUAUACCUgUgUAgUUUACCUgUgUggUCUgUgUggUgAgCggUAUCCUCCUUCAACCAAUggAUCCUCUUUCAgACCAAAAgACUgUUACAAAgAAUgAACUgUgUAUUUAUUUUUgUAUUUUUAUCAUUUAAAUUAUUUAUgCAAUCUgUUUUUUUUUUgUAgAAAAUAAUAgCCAUUAUUgUACUAUAUgUAAUUAUAgUgAAUUUCAUACCgAAAAAAAAgAgUACAUgAAUAAAgUUAAgCAACCUAUAUAUgUgAAAAgAA |
|  | *fgf23* | NM_001009564.2 | CUUUACGCGCUCUGUCAAGAUGCGUUGCGCACUUUCCAACCUGCACAUGCUGCAUUCAUCCGUCCUCGCGCUGUGGUUCACGGCUCUCCAGGGACUCAGACCUGCAGAUGCGGCCCCCAAUCCUUCUCCGCUGCUGGGCUCCAACUGGGGGAACCCGCGGAGAUACAUCCACCUUCAGACCACUUCAGACUUAAACAACUACUACCUGGAGAUCAGCCCGAGUGGACACGUGCGCAAAACUACAAAUCGGGGCUCAUACAGUGUAAUCUUAUUGAAAACAGAAAGCAGAGACCGUCUGGCGAUAUUUGGAGUGAAAAGUAACCGGUUUUUGUGCAUGGAUACAGGAGGAACCCUUUUCACAUCUACGAUCUGCAAUAAGGAAGACUGUCUUUUCCACCACAAACUGUUGGAAAACCAUCGUGAUGUGUAUUACUCCACUAAACACAGCAUACUGCUUAAUCUGGACGGGGCCAAACAGGCGUUUAUAGCGGGACAAAACCUCCCUCAGUCGUCUCUCUUCUUGUCGGAGAAGAACACAGUUCCGCUGGAGCGCCUGCAGCACCGGGAGCGCAGGAACCGGCAGGUGAACCCAACAGACCCGCUGAACGCGCUCCGGUACGCGGAGGAGUCUGAUUCCAGAGCCGCGCAGGAGGAUGAUGGAGACAUGGAUUUUGAGCCCUCAGAAGGUCAAAACAUCUCUAGAGAAACCCUUGUUUCCCCUUCCGAUGAUGAUCCAUGGGAUCUUCUGCACGACACGAGCCCUGGAAGUCCUCGGAUUGCAGCAAUUGUCGGAUAAAUCCGCCAAA |
|  | *Col1a1a* | NM_199214.1 | AUGUUCAGCUUUGUGGAUAUUCGGCUGGCGCUGUUGCUCAGCGCAACGGUGCUUUUGGCAAGAGGACAAGGCGAGGACGAUCGUAAGUAUAACUUUUCUAACUUUCCAAGCUUUGCUGGAGGGUAGUGGGUGCAAAGUGAAGAGGCAAGGGUAUUUGUGAUGCUGUGGAGGGAGGCAGCAAUAGAAAUAAUUGUGUCAUGAAUUUUUUUCCUAUGAGGCAUUGUCCUGUCGCCUGAAGCAUAGUUUUUAUAUUCUUCUGGAAUGGUGAAGAACUUUUUGCUUAGUCUGGAUGUGCAUUAGACCAACUGGGCCUUUCACGUUCUGCAUGCGUGAGCCUUUGGAGACAAGUUGGCACAGGUUUGGAAACUCUCCUCCAACAGCUGCUAAAAGAGAAAAGCAGACAGGGGAAAGAUCUGAAAGUCCAUAAUGUGCUACAUUUGAAUAGAAAAUGUUAACAGUUUCUUAAGGUGUAAAAUAUAAAUAGUUUAGUAUACAGUUAUAGAUUUGACCAGAUGUGGAAAAAAGAAGCUUUAGACAUUAAACAUGUUAGAUUUAAGUACAAAUAAUCAAAAUAGCACAUCCAUCAAAAAAAGUUUCCCAGAAACAAAAAGAGAUGAUCUCAUUCUUGGAUGAGCAUCUCUCCGAACUCCAUAGUGAAUUCUGCUGAUUGCGUUAAAACAGAGCCACUCGGUUUUCUGACCAAUAGGACAGAGAGAAGAGUAGUUUAAAUAUAAGUUAUAUAUUUAAUGUAUAUUCCAGACCAAUACUUUUUUACCAGUAUUAGAGCUGAAGUGUUUUGGACGGUGUCGCGGCGCCACCUGGUGGUUAAAUAUGCAAUGACACCAUAUUUUGAAGAUUCCCCUCCCCCAUCCCUUUCACUUUUCCCCCCUCUGUUAUGAUGAUUGAAAACACAUGCGGACCCUGCAGACUUUCCACAGAAGGAAAACGUUAGUCGUUGCCAAGCCGACGGUCUUCAAUUACUUCAUGACGGUCAGCUUUAGCAGACGUGGAAAUUUUCCCUCAAGCUUUUCCACCAGAAUAAUGGAAUUUAGGCAAAAGUGCACUGCGCCACCUAGGGGGCAUUUUCAAACCAAACAGUUUAACCACUAAGGUAAAGGUGUGUGCGUUUAUUGACGCAAUGGAUUGUUUUCCCACGUGUUUGCAUAAUGUUUACAAAACAAAGCACACAAGAUAAGUCCUCAUUUCAGUAGAUGCUACUAAAUAAUUUCGAAAAGUGAUAACAUCACAGUUUAGAGGUUAUUUAAAAUCUGCAAAAGUGCAUUAUAAGCAUUUCCCACCCUGCGUCACUCAUUUAGCCUGACUACUGUAAAAAAGAAACCUAUUGACCUGAAUUAGACAUGAAUAUCUAAUAAAUUAUUCGACAUACUCGAAUAUUAAAUGCCAUCUUUUCUAAUUUCAUACUUAUAUAAGUGUUUUUCUCAUUACUCCUGCAGGCACUGGCGGCAGCUGCACAUUGGACGGCCAGGUCUACAAUGACAGGGACGUCUGGAAACCAGAGCCAUGCCAAAUCUGCGUGUGCGACAGCGGCACCGUAAUGUGCGACGAAGUGAUCUGCGAGGACACAAGCGACUGCCCCAACCCAGUGAUUCCCCACGACGAGUGCUGCCCCGUCUGCCCAGACGACGGUACGACGCCUUGAUUCUCCACCUUUUCUUACCCUGGGCGACCCCACAUCCCACAUUUAAGAUAUCUGGGAAAUUUAUCACGGAUUCACAACAUUCUCUCCCCAUUGGAUUUUAAAAUGUGAUCUUCAAGCCACCAGCCUUAUGGGCACUCACUGGAUUAAUUUCUCUUUGGUUCUUGGGUCUAGUAGGCCUGUAAGUCAAAGGUCAAGGCCACAAAUCUAAUUGGGGAAAACCACAGACUCACUUGAUUAGAUGAUUGUUAAAUUGGGCUAUUGUUCUGAGAGAUAUUAUUGUUAUAAGAGUUAUAAUAGAGUUAUAAGAGACUCUCAUUUUAAUAUUGGUUUUAAUUUUUCUUUUUUUAUUAUUUCCUCAACUCUAUAGAUUUCCAGGAGCCCAGUGUUGAGGUAAAUGUUGUUUUACUUUUUGUUAUAAAUUACAAUAUGCCUAAAAUAUAUUUUCAGUUCAAUGAGGAUGUUGAUUUGUAGUCUGUAAUAUUUUAAUAAGAAUACUAUACCUAGGUACUACUUCAGAUAGUUAUUUUGAUCUUUAUCGGGGAAAACAAAUCCUCAUGAACUGCUUUGUGUUCAUCACUCUCUCUUAGGGACCUAGAGGUUCUCCCGGUGACAAGGGUGAGAGGGUAGGUUGAUAUUUCUGACUUUUUUGUGCAUCUGCUUAUUGUUUUGUCAGAGGUUGUGUUCAAUAGCUGUUUGUCUUUAAAAUCUUGAAGACCUAAACAAAAAUUGUGAAUUAGAUCUCAUGAACACUACAGAAAACCGUGUUGUAUUCCUUUUAAGUUACUUUUGAUUUGAUCUAAAAUGAUCUAAACUAAUUUAUUUAGCUACUCAGUUCGUCAAAAUGUGUCACUGAGGUUAUGCAGGGCUUUUAUUAUGACACUGAUACAUAAUCAUAAGUACUCAUAAGUCUACAGUACUAAUAAUACAACCUAUUUCUAACAAAAAUGUUAUACAUGCCAAUGCUUUAGUUUCUUUUAUUAUAAGGUAUUUGUAAGGCUUUGAACUCUGUGAUGAGGCACAGAUGUUUUGGUCUGAAUGCAAUAUAAAAAGACAACAGAAUUAUAUAUCGUCCCACCACUUUCCACUAAUUAUACCUUUACCAAGCCAAAAGGUCUGCUCUAUAGAUGUGGUGCAUCAGUUUGAACUGUAAGGGCUUUGAUGUGAUGUCGUCAUCAGUGCAUAGACCUUUCUGUAUCAAAGUGUAGCAUCUGUCUCAUCUCCUUAAAUCCUAAUUUUCCUUUUUCUUUAUGUUAAACAGGGUCCUGCUGGCCCUCCCGGCAAUGAUGGAAUCCCUGGACAGCCUGGCCUCCCUGGGCCUCCAGGCCCUCCUGGACCUCCUGGCCUUGGUGGAGUAAGUAUCUCACCCUAAAUAAAGGACAUUUCUCUUAGAGAAGAAACUGUGACUAUGUGCCUCCAGGCCAGGUGCACAACUUCAGCAGUUGGUCUAGGCCAGAAUGUGAAAUUAGAUCACAUUAAAAGACCUUCAGGUCAAAGGGCAGAUGCAUUUCAGCACUAUUAACGUGGACAGCGCCCUCAGGCAGUAGCCACAGUUGCACACAAGAAUAGAGGAUUUCUAAGAUAAUCCCCUGAACAUGAUCUAUAUAGAUAAAUCUUUAUGCCGGUUUCUGUACAAAUUACUGACAUGGUCUUUCCUUUUUCUCUUGUGCCCCUCAGAACUUUUCUCCUCAGAUGUCUGGUGGCUUUGAUGAGAAAUCCUCUCCAAUGGCUGUCCCAGGCCCCAUGGUAAAAAAAUCCCUGAAUGCUUAUUUAACAUAAAUAUUCCUAGGCAUGCAUGCACAUUGAUGUGCAUCAUUUAAUGUGAGACAAAUAUCAGUUCUAUAAAUACCCAGUAUGCAAGGUUCACCUAUUUAUCUUAAUAUAAAAUCACCAUAGAGUGAAGUUUUAGCACACACAGUCAAAUCAGCUGUUAAAAUGUUGACUCUGUUUUUUUUUUUUAAAAGGGACCUAUGGGCCCCCGUGGAGCCCCUGGACCUCCUGGACCUUCCGUAAGUACAGUUCUUGAAAACAUUUUUAAAAUGUAAAGUGCUCCAUAGUGUAAUUCAUAAUUAAAUUAAUUUGGGGUAUUAAGUAUUAUUUUGGAGACUGAAAUUUGCCUUAUGCAUCUUUCAAAAAUGAAUAUAUCAUAUACAAAUUGAAAUCAACAUAUUUUGGGGACAUCUGAAUGAUCUCUUCAUAUUAAGAUUCACUCAGGAAAUCUAACACAGCUACUUCCAUUGUUUAGCAUGUACUAGCACUGUGAUUCCUGCUUUAAUUUGUGCACUUUGUCCACCUUUACAGGGACCCCAAGGAUUUACUGGUCCCCCUGGCGAGCCUGGUGAGGCUGGUGCUCCUGUGAGUAUCUAAAUUAGUUAUCUGCAAACUCAUCAACCCUGCUUCAUUCUGUUCAUUACAUAAACAGUUAGAAAAUUCCUUAAACAAUGAACUGACCAUGAAAUCUGAGAUUGCUCUACCUGUUUACAUUCACCUAUAUUGAUAGAACCUUUACCUACUUGCAUAUGUGCCCGAGUUUGAGUCAUUUGGGUCAAAUGAGGUCAAAGGUAAACAAAGUUCAUGUAACUUGACCUUAGCCAUCUAGACUGCUUUUCUACUGUAUGGAUGCAUGGACAUGCAGUCUGUGUCUCAACAGAGCUUUCUUUCUGACCUGGCAAGAUUUUCACUUUAUUUCAUCUGGAUAAUUCAGAACUGCCAAAGUGUGACUCACUCAUGCCGCCACACAGUGACACACAUAAAUACACUCACAUGGACACUGAUGUUUUUUCCUAACCUAGACUUUCUUUCAUUCUGUAGGGUCCAAUGGGUCCCCGUGGCGCUGCUGGUCCCCCUGGAAAGAAUGGAGAGGAUGUAAGUAACCCAUAUAAUUUGUUUAGUGUUGUUUGAGACAACAUCAGAACAACCAUUUUUAACUCCACUAGUUUAGGCAGGGUUGUUUUGUUGUUAUAUAGAUUUUAAGGGGACUAGGCAUAACAUUUCGUAACAUCAAACAGAAACGUUGGUGAAAUGUUCCUCAUAGUUUUUUCUCUGAAUGUGUAGGGUGAGUCUGGCAAACCUGGUCGCCCUGGUGAGCGCGGACCCCCUGGACCACAGGUGCGUUUUAACAAAAAGAUAACAUGAAAAUAAUCAAAUAUACUAAUAGAAAAUAUAGGCACACGGUUAUAGGACACACAAAGGAUAAAUGGGUGUAAAAUGCAUCUUUCCUUUCUCUUUUUAUCAAGGGUGCUCGUGGAUUCCCCGGAACCCCUGGACUUCCAGGCAUCAAGGGACACAGAGUGAGUUUGCUAGUUUGAGGAAAUGGUCAUUUGACAGAUAUAAAUCUGUUUUCACAUAUUAAAUCUACUCCAAUCUUCUGUACUUUAGGGAUUCAGCGGUCUAGAUGGAGCUAAGGGGGAUGCUGGCCCUGCUGGACCUAAGGUAAAAUCUAUUCUUAUUAAAAUCAUCAUCAUCACGCUUUAAAUCUCAGUAAGCAGACCAGCUAACAUGCGGCUUUGUUUAUUAGGGUGAGCCUGGUGCACCUGGUGAGAAUGGAACUCCUGGUGCCAUGGUAAGUAAUAGUCAUCCUUGAUUGGCUUAUUUAAGGAUGACUUCAAUGCUAUUUUGCACAUCUUUUCUAAUGUAACUCUGUUUUUCAGGGUCCCCGUGGUCUGCCUGGCGAGAGAGGCCGUGCUGGUCCUCCUGGUGCUGCUGUAAGUAACCUUUCAACCCUGAUCUAAUCUGUACUUUGUGUCCUAGUAUAUGUUUACUUGCAGGUUCUCACCUUCCUAAAUAAUAUAUGCAUGGAAAUUAAACCCUUCACAAUAGCUUAACAGCCAUUUGGAUAUGAUUUUUAUAAGUACAUAUAUGUAUUAAUAUAGACAUUUUGUUUAAAAUAUAUAGUGUGCAUAUUAUGUUUUGUAAUGCCUACAAAUAAUCACACUCAAACUAAAUCUUUCAGGGUGCUCGUGGUAAUGAUGGUGCCGCUGGAGCUGCUGGUCCUCCUGUAAGUAGCUACAAUAUAUAAAAAUAAUAAUAAUAGCCUGAAAAAUUAUGAUGACUAAAAUAUAUUUCUUUAAUUCUUUUGCUUUAGGGCCCAACUGGCCCUGCUGGUCCCCCAGGAUUCCCUGGCGGCCCUGGAUCUAAGGUAAAAACAUAUAUAUUUAUAUUUGUUCAUGAUUAAACAUAUUCAAACUCAAAAUCAAACCAAACAAACUCUAGAGGACACAAAAUGUCACUGUAGUUGGAUCAAAGGCACCAGAUGUCUGGAUUGUUGGCAAUUUUGUUAAUCAUCAUGAUGAUGUAGGCCAACAAUGAAUGAAUGAAUCCUUGGCAAAUAUUUGUAGGUCAAAUCUUAGAGUAACAUAUUUAUUAGCUUAUUACUUGUUUGGUUUUAUUGGUUAAUAUAUAAUUAACUUCAACCAUUUACAACAGAAAUUCAGGUGUUCAGAAAUCCUGACACAAAUACCAUGAGAAUCUUUAUUUUUAUGCAAUUCUAAAUUUACAAUAACUGUUGAGAUGCCCCUUGAAGCAUUUCUUAGGUGUUUUAUUAGGUGUUUUUAUUGACCUAUCUGUUGCAUUUGCUUUCUUUAGGGAGAGGUUGGUCCUCAGGGAUCCCGUGGUGCAGAGGGACCUCAGGGAGCCCGUGGUGAAGCUGGUAACCCUGGACCUGCUGGUCCUGCUGGUCCCGCUGUGAGUGAAUCUUAUUAUCCAGAUUUGUUUAAGGUGAAAUUAUUAAUUCAGUUCACUCAUGAACACAUUGUCUACUUUGCUAUUUUUAUUGUAGGGUAACAACGGAGCUGAUGGUGCCCCUGGUGCCAAGGGUGCUCCUGUAAGUAUUACUUUAUUCUUUGAUGGUAAAACAUUUUCGUUUUAGGCAGUUAUGUUUCUUCAUGGACGUGGGAAGAUGUUUUGGGAUAGGGGUGCUGUAUUAGGGGGGGGAAUUAUUAGGUGAUGAUUUGUCAAAAAAUUGACAAAAACACUGUAGCCUUUACAAGAUUGUUGAAGUCAAUUGAGCAAAUAUUGCGGUACAUUUCACUCUAGAUCUGAUAUAAUGGUAAUAAGCCUGAUAUGUUAUGCUAUAUUUUUGAGUCUAAACUUUGUGUCAGUGAGCCAUCUUGGCAUAUUUGCAAAUAGAAUAGUAAAAUAUAAAAAAAAAUUAGAUUUUUCCAUUUCCAAUGUUGCCACCUUGUGGGAAAAAGCAGUUUUACACUGCAUGUUUGAGCUUCAUAUGCACAAUUGGAGAAAAAAUAGCCUGAAGUAUAUUAUAAAGGUUUAAUAACUACUACAAAUUACAGUUAAAUUACUCAAGAUUUACUGAUUUUAAAUGUAGCCAGCUGAUUUCUGCAUUUAAAGAAUCACAGUGUUUACAGUCCCGUAAAAACAGGGGGGUGCCGCAGCACUUCCAGCACCCUUACUUCCCACACCCCCUGUAUUCCCACUUCCUUGUUUUGAAUGUUGAAAGUCACUUGACUCCCUCAUUUUUGCAAAUCUCUGCUGCCACCUAUAGGGCGCUCCUGGUAUUGCUGGUGCUCCUGGUUUCCCUGGACCCCGUGGUCCUCCUGGAGCUGCUGGAGCGGCUGGUGCCCCUGGCCCUAAGGGUAACACCGUAAGUGCACAGAUCUUCUCGGAUUCCACUACAUUUUUUUUUAAACAAAAUGUGAAUAAUUUUACACAAUUACUUCUUACAAUUUUUAGGGUGAGGCUGGUGCCCCAGGUGCCAAAGGAGAGGCUGGUGCUAAGGGAGAGGCUGUAAGUAUAAACUUCACUUAAAAUUAAAUACAUUGCCAGUUACUUUGUCAUUUCUAAAACUCCAUUUUCUUAUAUUGCAUUUUCAGUUCCACGUCUUUGUAAAAACAUUGUCCUUUCAGAAAGCAACUCUGCCAGAUAUUAUACUUCAAUUCAUAUCUAAUUCCAGUCUUCAAUUCUAACCAUCUUUGUGUGUUUACCUAGGGUGCCCAAGGAGUUCAGGGUCCCCCUGGUCCCCCUGGUGAGGAAGGCAAGAGAGGACCCCGUGGUGAGCCCGGUGCUGGUGGUGCCCGCGGACCUACUGGUGAACGUGUAUGUACCCACAUCAAACCAAUCCAGAAAACUGAAGACAUCAGUAGGAUCCUUAAAAGUAGCCUAGACAAUAACACUUACACACCUGUCUGUGCAGGGUGCUCCUGGUGCUCGUGGUUUCCCUGGUGCUGAUGGAGCUGCAGGUCCAAGAGUAAGUCUACAGAACAUACAAAAUAAUUUAGUUACUAACUAAGGAAAGGAUAUGUUUAGGAUUUAAUUGUAACUCUUUUUGAUCUGUGUAACAGGGUGCCCCUGGUGAGCGUGGAGGCCCUGGAGUUGUUGGACCUAAGGGUGCCACUGGUGAGCCUGGCCGCAAUGGUGAACCUGGUAUGCCAGGAUCCAAGGUAAGGAAGUUGGCAUUCCUGAUUAUAUAUAACAGAUAUCAACUGGUUGUGUUUUAGUAGGCCUGCUUUUGACCCCUCUCUCUUCAUCCCUACUACAGGGUAUGACUGGUAGCCCUGGCAGCCCUGGACCUGAUGGAAAGACCGGACCUGGUGUAAGUGCCCACUACACCAUAACAGAUAACUCAGUGCAUGGUGAAGAUGUCUAGUUUACUACUAUUAGAAUCCAGGGUCUGCAUUUUAACAACUCCAACAGCUUUUGACCACUUUUAAUAAAUAAAAAAAAUCAUAGGAAAGUGAUUUAAAAAGUGUAAUUACUUCAACCCAUGAUCAUAUUCAUGUAUUCCUCUAUGAUACAAUAAUUCCAGUACAUUCAAGAGUUCACACUAAGCUGAUGAUUGAUUAUAAGCAAAUUUGGCAUGCUGUCCCGGGAGAGAGCCGUGAGCUUAAAGGUUCUUGAGCCCUGGGCUCCCUCCCGUUUGAAGGGCGAGAGGGGAGCCUUGAGCUCAGGUAGAUCUUGACAGCUCCCCCUUUGAUAAAAACUGAUGACUAAAAUUGAAUGCUAUGAAAAGAUAUGCUGCAAGGUGAGGGAUUAACUGUAGUGCAAAAUUUAGAUUUAUCAAUUUAGUUGUUGUGCAUGUUUUUGGAAUGUGGGAGGAAACCGGAGGACCCGGGGAAAACCCACGCAAGCACGGGAAGAACAUGCAAACUACACAUAGAAACGACCACCGGCCUGUUAAAGUACUAGAACCGGUGACGUUCUUGCUGUGAGGCGACAGUGCUAAUCACUGGGCCACCGUGCUGCCCUAUGGAGGAAAAGGUGAAGAGGUAGGGGUGGAAGGGGGGAUUCUUCAAAUCGAAGAUGACUGUUGUGAAAAAACCCUGGCUCUUUAUAGUGGGUUAGGAAUGGCCUGAUUGGUGGAUCAUGCAUGCUAAUGCAGAACCAGCCGAGUUCAUUAUCACGUGAUCCUCUCGAAAUUAGUUUAUGAAUAAACUUCACUUCAGUCAGGCAUAUAAACUCAUAUCCAGAUAUUCACUCAGGGUGAAAAACGAUGAAAGCUCACAAAUCUCUUUCUUUUAUAUCUGAUAGGGUGCUCCUGGACAAGAUGGCCGCCCUGGACCACCUGGCCCUGUUGGAGCCAGAGGACAGCCUGGUGUCAUGGGAUUCCCUGGACCUAAGGGUGCUGCUGUGAGUGCCUAAAUAACAAGAGGCCUUGUUCAAUAUGCAUUUGUUACAAAAAGCUUUCUACAUUUGAUGCUUAUUUUUGGCCUUUUGUGCAGGGUGAGGCUGGCAAACCUGGUGAGAGAGGAGUGAUGGGUGCUAUUGGUGCCACUGUAAGUAUCUUUUUGUUUUUGAGUUUACCUAUGCUUUUGGUUUCAUAGUCUCUGCAUCAGAAGAGACCUUAUUCUGUUUUGUUUUAUUUAAAACUAUAGGGCGCUCCUGGUAAGGAUGGUGAUGUUGGUGCCCCUGGUGCUCCUGGACCUGCUGUAAGUAACUCAACAGUAUUUCAUAAUAUAUUAGCUAAAUAAUUUCAUGUUAUUUAACAUACAUUUAAUCAGUGUUCAUUCUAAUCCAUUAUAUAGGCCAUCAAACUUAUGUUUGUUUAAAUACAACAUUACAAUAUAUAGCUUGAAGUUACAUUCUUCUAUAAAUGACUACCUUAUAGUAAUAAUGCUCAAGUGUAUUUAUUUUAAUGAACUACUUUGUCUAAAGUCUCUUGAUUUUGUUUGUGAAUAGGGACCUGCUGGUGAGAGAGGUGAACAAGGAGCUGCUGGUCCUCCUGGAUUCCAGGUGAGUGUGUUUAAUUGUUUUACCCAUCCCAUUUACACUCUAAAUGCUGUAUUUUUGUCAUUGAUGUUGAUUUUAGAUUGUUCAUAUCUUUGUUGUGCAUCUGAAUGACAGGGUCUGCCAGGACCUCAGGGUGCUACUGGUGAGCCAGGAAAGUCUGGUGAGCAGGUAAGUUUAAAUGUAAUUGCUGUCUAUUGUAUGAUCAAUGAAGCAUUGCUUAAAAGGACACUACACAUAAACAUGGGCAACUUUGAAAGCCUUAUCAAUACUAACACAAUACUAAUUUGUGCUCUUUGAUAUAAUACAGGGUGCUCCUGGAGAAGCUGGAGCUCCUGGACCUUCUGGUUCUAGAGUAAGUAUCUACCAGAUGAUCAGCAAAUGGCGAAAAGCUCAUAUAUGAGAGGGACCAGAACUAAUGGCAUCUGUAUUCAUUUUUACUCAGGGUGACAGAGGAUUCCCUGGUGAGCGUGGUGCCCCUGGCCCUGCUGGCCCUGUUGGUGCCCGUGGUUCUCCUGGUUCAGCUGGUAACGAUGGUGCCAAGGUAAGUCUCAUCUCUUCUGACUAUUUUUAUCAUGCCAGACAUUUACAGUGAAUGACUAAGAUAAAUCAAAGGUUGCCACCAAAUGUGAACCAAUCUCCUGGAAAGUAUGGCAACAAAUCCUAGUCUGUAUCAACAUUAAGAUAACUUGUUUUUCAGGGUGAAAGUGGUGCCGCAGGUGCUCCUGGUGCCCAGGGUCCUCCUGGACUUCAAGGAAUGCCUGGAGAGCGUGGGGCUGCUGGCCUCCCUGGUCUUAAGGGUGACAGAGUGAGUAUGACACAAACUGAACUUUGAACAGUUUGUCUUUGACAGAAAAUAUUGUUCCAACAUACAGCGAACAAAGUUGUCAAAAUUGUUGAUCUCUUAUCCCAGGGUGACCAAGGUGCCAAGGGUGCUGAUGGUGCUGCUGGUAAAGAUGGCAUUCGUGGUAUGACCGGACCAAUUGGACCCCCCGGACCUGCUGGAGCUCCUGGUGACAAGGUGAGUGUCUUUUGAGCUAGAUUCUAUAAAUCCAUACUAAGUGAUGAAACAUCUUGAAGUUGAAGAGUAUAUUUAACCAUGGUUCUAAUAUUUGUUUGUAAACAUAGGGAGAAUCUGGUGCUCAAGGACUUGUUGGACCUACUGGUGCCCGUGGACCACCUGUAAGCACAGUCUUGGCUUUAAGACAACAAACAUUUAGGUAUUUCACUAGAAUGGUCUGAUAUCAUAAUCUGUUAUUACAUAUAGGGUGAGCGUGGAGAGACUGGUGCUCCUGGACCUGCCGGAUUUGCUGGACCUCCUGUGAGUAACAAAUUCUACUACAUCUAUAUCCUUUUAAUGUUUUUUACAGCCCAGUUCAUAGUUCAAACCUAAUUUCGUGCCAAACAGGGUGCUGAUGGUCUGCCUGGUGCCAAGGGAGAACCUGGAGAUAAUGGUGCUAAGGGUGAUGCUGGUGCUCCAGGACCUGCAGGAGCAACUGGUGCCCCUGGACCUCAGGUGUGACCAUAAUUUCAUAUAAUUUCCUCAUGCUCCUUUUCAGAUAUGUGCUUUUCUGGACCUAUAAAUGUUAUCCUACAUCAUAAGUCAUGGUUUUCUUUAGUAAUGUAGAAAUGCAAUUUUUUUAAUCAAAUUGACUUGUGCUAUGCAGGGUCCCGUUGGUGCUACUGGACCCAAGGGUGCCCGUGGUGCUGCUGGUCCUCCUGUGAGUAUGACAACCACAAUUUCAUGGCGUUUGUUAGCACUUAUCACAAUUUUCUUCUUUCGAAGAUUCUUUACAUAUAUAUUUUUAUUUAGGGUGCUACUGGCUUCCCUGGUGCUGCUGGCAGAGUUGGACCUCCUGGCCCUUCUGUAAGAACAUCUACAAUAUUGAUUUAGAUAUGCAUAAACUGCUCUCUUGUACAAUUAACAGUGAGUCACUUAUUACUUGCUGUAUUUUUAGGGAAAUUCUGGACCCCCAGGACCUCCUGGACCCGCUGGUAAGGAGGGCCAGAAAGGUAACCGUGGUGAGACUGGACCUGCUGGUCGUACUGGUGAAGUUGGUGCUGCUGGACCACCUGGUGCCCCUGGUGAGAAAGGAAAUCCCGGAGCUGAGGGUGCCACUGUAAGUCAUUCUGAUCAAUAAUUAUACUUACCUUCAGGCCUUGUCAAAUACAUCACUGUAAACAAAAUUGUAUAGAGCAAAUUUCCAAUAUCGCCUUGCAAAAUGAUUCAUAUUGCUUAACACUUUCAUUUUGAAUGCUUUCAUAUUGGUAGAGAGUGAUGUGUCAUGUUAUCCUUGGUUCAAAAUCAACAUUUUAUCCCAAAUCACAAAAUGCAGAUGAGCUUUAAUAUGGUUAUUUUAAACAAGUCUGUGGAUGUGAUAUUACUGGACUUUUGUGCUUGUUACAGAAUAGAUUAUCUUCUAGAGAAAGUAAAGAUAUUACCCUUUGCUCCUCAUCUUGAUCUCCAUGCUAGUAUAAAUGAGUGGAUAUUUAUUUCAAACAUCAUUUCUAGCUGUAAGAACACACUUUGUUGUGCAUUUUCUUAUAUUUCACUGACAUUCCUCUUACUUUUUCAGGGCCCUGCUGGUAUCCCUGGACCUCAAGGUAUUGGUGGUCAGCGUGGUAUUGUAGGUCUCCCUGGACAAAGAGGCGAGCGUGGUUUCCCCGGUCUCCCUGGCCCAUCUGUAAGUUUUCUGGCUCUUUGAAAUCUCAGUUGAAAAUAGAAAUACCUACAUACUAUGUCUCUCUUGUCUUGCUUGAAAGCUGGUGCCUCAAGGAUCAAUCAUGGACAAACUUUCAUAAAGUUUCCUUUAUUAUGCAAUCUAGGGAGAGAUUGGCAAACAGGGACCUUCUGGUCCAUCUGGUGAACGUGGACCUCCUGGCCCCAUGGGACCCCCUGGACUGGCUGGACCUCCUGGUGAGCCUGGUCGUGAGGUGAGGAUACACACCUAUACCCCAGAACUUGCUGAAUUUCAUUGACAUAAUUGAACUGAAAGAAAAGUAGUACUGCCUUGUUAACACCUAUAUUUUUCUUUACAUUAGGGUACUCCAGGAAAUGAGGGCUCUGCUGGACGUGACGGUGCUGCUGGCCCCAAGGUGAACUACAGUCUUCAUGGUUUCCCAUAACGCAAUGCAUUCAGUUAUCAUAGCACUACACAUUUGGCUGUCUCAAAGAUGGUGUCCUUUCAAAAUUCUGUUCUUCAUCUCCAAACAUAGGGUGACCGUGGUGAGACUGGCCCAUCUGGCACUCCUGGAGCCCCUGGACCUCCUGGUGCUGCUGGACCUAUUGGCCCUGCUGGAAAGACUGGUGAUCGUGGAGAGACCGUAAGUGUCUCAUCUCCAAUGAUGAAUCAGGGAUGGAUUGUUUGAGGGGAAUUUCAGACUCAUCUGUAGUUGUUUUUUUUACUUGUUAGGGCCCUGCUGGUGUCCCCGGCCCUGCUGGCCCCUCUGGUCCCCGCGGACCCUCUGUAAGUUUUGACUCAAACCACAAAAGAGUUGCCCAAGCAUACUCUGUAUGUAGCUGUAGUAUGAACAAUAUUGUGUUCUAAGGUGUCAUGUCUUUAUCCAGGGACCCGCUGGAGCUCGUGGAGACAAGGGUGAGACUGGUGAGGCUGGUGAGAGAGGCAUGAAGGGACACAGAGGAUUCACUGGAAUGCCAGGACCCCCUGGUCCUCCUGUGAGUGAUUCUGUUUGGAAUAUGGAAUUUGCAGUAUAUGCCUUGAUAAUAAAAUCUGUAGAUUGAAAUCUGUUUUCAUUUCCAAUAUUCCUGUCCAUAGGGACCCUCUGGAGAGUCUGGACCCGCUGGUGCUUCUGGACCCGCUGGACCAAGAGUAAGUGUUAUUAUAAAACUUGCACUACUGCUGUUUCUCUGUUUUGAUCGAAUACACCAUGUCCUUAGUUCAUUCUGAUUAAAUUUGAAUGUUUCUCUUAUUGUGUUAAUAGGGCCCAGCUGGAUCUGCUGGAUCAGCUGGUAAGGAUGGUAUGAGUGGCCUCCCUGGACCCAUUGGACCUCCUGGACCCCGUGGUCGCAAUGGAGAAAUUGGACCAGCUGUGGGUGAUUCUUUCCUUUUUUACAUAAUCGUGCACUCAAUAAACUAAAUAUAUGCAUUAACACAAGCGUAUAUGGUUAUAGGGACCUCCUGGACCUCCCGGCCCCCCUGGAGCACCUGGACCCUCUGGUGGUGGAUUCGACAUUGGCUUCAUUGCCCAGCCACAGGAGAAGGCCCCUGAUCCCUUCCGUCACUUCCGUGCCGACGAUGCCAAUGUGAUGCGCGACCGUGAUCUUGAGGUUGACACCACCCUUAAGUCCCUGAGCCAGCAGAUUGAGAGCAUCAUCAGCCCAGACGGCACCAAGAAGAACCCUGCCCGCACUUGCCGUGACCUGAAGAUGUGCCACCCAGACUGGAAGAGCGGUAUGUUACUUUUCUUUGUUGUACAACAAUAGAGAAAUGUCUCAUUUAAAAAUUUGUAAAUGUAUUGCCUUGCAUAGUUUAAUCCCAUUCCUCUCAAAACCAUAACACAGGCGAGUACUGGAUUGACCCUGACCAGGGCUGCAACCAGGAUGCCAUCAAGGUCUACUGCAACAUGGAAACUGGCGAGACUUGCGUCAACCCAACUGAGUCUGCUAUUCCCAAGAAGAACUGGUACACAAGCAAGAACAUUAAGGAGAAGAAACACGUCUGGUUCGGAGAGGCCAUGACCGAUGGCUUCCAGGUGAGGAUGUCAACUAGUUCAAAACCUACAACAGAUUUUGUCAUCAAGAGUCAUAUUUUUGCAAACCUCAUACUUUCCUCUUGAAACCAACAGUUCGAGUAUGGCAGCGAGGGCUCCAAGCCUGAGGAUGUCAACAUUCAGCUCACCUUCCUGCGCCUCAUGUCCACUGAGGCCUCCCAGAACAUUACAUACCACUGCAAGAACAGCAUUGCAUACAUGGACCAGGCUUCUGGCAACCUGAAGAAGGCUCUUCUCCUGCAGGGCUCCAACGAAAUUGAGAUCAGAGCAGAGGGCAACAGCCGCUUCACAUACAGUGUCACUGAGGAUGGUUGCACGGUGAGUUGUCUGACAUUUUAUUGGUUGAUUUGCCAUCUCAGCAGCAAUGUUUACUGAAUAUGAAAGAUAAUGCUGAAUAAAUGUGAAAAAUGAGAAGUGUUAAGUACAUUUUCCUACAUUUAUUAGCAAUAGCUUUUAUCUCAAUCAACUUUUGGAUGGUAAUAAAAGCAGAAGCGAUUCAUCCAAAGGAAAUCAAGCUGCUAUUGAUUUAGCAUUGAUAGCAUUAGCAGUGGUGUAUAUAAAGGCUCUAAUUCUGUCUCUGUUUUGCUUGUUUCCAUCUCUACAGUCGCACACCGGUGCAUGGGGCAAGACAGUCAUUGACUACAAAACAACGAAAACAUCCCGUCUGCCUAUUAUUGACAUCGCCCCUAUGGACGUUGGUGCACCUAAUCAGGAAUUUGGCAUUGAAGUUGGCCCAGUCUGCUUCUUGUAAAAAAGAAAUCUGGACUUGAGAUUGUUUGUGUGCGUGUGUGUUUUUUUCUAGCAAUCAUCUCUCUAAAAUCUUUUCUAUGCAUUAAAAUCAAACCCUAGAAUCCAAUUUGGCUGUCCAAAUGGUCCACUUGCUUAGACCUGCGCUUCAGAAGACAGUGGUUGUGUGUUAUCAGGCUUUUUUUGGACCACUAUUUUGUCACCACCCACACUGAGGAUACUUUAGAAGACUCAUUUGUUUCACUGGCAACAAGAAAAUAAUAUAUACUUAUAGCGUGUAAAAUCCCCCUGGAGUUUUAAAGAAACAUCAAUGAUACAAGUGACUUUUUUCAACCACUGAGGAAGGACAAUGAAAGAGCGAAACCAAAUGCUUUGUACCAUGUUCAGGUGUUAAAUAAAUCUGUAAAAUGACAUUUAAGUGUCUCUGUGUUAUACCCAUUAGGACCACCGGGUGCCGAAUGCCAACGUUCCUCUCAAGUCACACGAGUACAUCUAGUAGAGACAGGGGUUCAACUUUGUUUUGAUGUUUUAUGGCUACCUCAAAAAAGAAACCUGCGAUUCCCAGUGUUGACCAACACCAGGUCAGGAUUUUACUUGGCAGAAGUAUUGUUAAGAGAACAAGUGACAGCUGUAAAGGUGCUAUUUCCUCAUGAGGUCCUGUUAGCAAGAAAUCAAGGAGUUUCACAAAAUGUAUUGUUCGUCGCUUUUGCUCACAGGGCCUUUGGUGCUAAGGAACCCCAGCAUCUUGCUGCCCAGUUGUAACAGAUGCUUCCCUUUCUGAAGUGAAGAGCCAGACAGCAGAGCUUUUGUUAAAAAGCAGUACUGUAGCUGUGAUGCAUGCAGUCUUUUCAAGUGUAGAAUAAGCAGGUGGAGUCUGUUUUUAUUUUCCAAACGCCUUUUGACACUUUUUGGUUGUUGUCUUUUGGUUUCUUUAUAAAAUAUCCCCCCACAAAUGUCGUUCAGUUGCUUCCAGCUAUUCCCCCUACUUCCCUCCCAUCCUUCUGUAAUCUGGAUUUGUUUUGUUCUCACCCUGGUUUUUUUUUUUAAGCGUAAAACAGACAAUCAAAUAGAAAAGUCCUAAAAUCAUGAGAGAAGCACACCUUAGGGGGGUAAAAACAAGAAGAUCUGUACCUAUUUUGUAUAUGUAUAAUAAUUUGAGAUGUUUUUAAUUAUUUUGAAUGCUGAAAUAAAGCAUGUUAAGUGGUCG |
|  | *trpc5a* | NM_001327747.3 | GUCUGACACCAUGACAACCGCAGCGCGCGAACGCUGGAAUCUCUGCUGCUGCCGCGAAGCCCAAUAUGAAGACAAGAUGCCGCCAUACGAGUGCGAGACGCACUUGCUUUUUUGAGAUUUUCUUCGCUCCGCACUUUGGGAAGAAGACGUCGGAGAUUUGCCAAACCACCUGUAUCUAGCGUCCGUUGCCAACGUGAGUCGAACAGCCUUUCCACUGGCCAAACCUCAGUAUUUCUCUGCGUCGCUGGGUUGAAGACGCUGGAAAAAAAGCCGCGCGUAUAGGAGCAAUGAAAUGCGCAUUGGAGUCAGGGAUGCUCCUCAUGGCGUUUACGCGCGGAGAGCCCACUGGGGCGUGUGUGCAGUGCGUGUUAAUGCAUGUGUGAGAGAGGGAGAGUGAGAAAGGAAAACAGAGGAAAGGAAAAGAGGCUUAUGUUCAUUCGCCGCACGCGUUCCUUUGGCACAACAUGGGGAAUAUGGGGAUCUCGUAAGGACGCCAGUGAUAAAUAGUAUUCCAGACUCUCCUGCCACAUAAGAAGACCACGGAACAGCCUCCUCAGAGACCCCUUGGUCGAUACAAGGGGGGAUUGAAUAUCCAUUAAGUAAGGCGCAUCAGCAUCCAUCAUGAAUCCCAUGUCACAUCUAUACUACAAAAAGAGCAGCUACUCUCCGUACCGGGACCGAAUCCCUCUGCAGAUCGUCCGGGCGGAGGUAGAGCUCUCUCCAGAGGAGCGUGCGUUCCUUUCGGCUGUGGAAAAGGGCGACUAUGCAGGAGUCCAGCAUGCACUGCGUGAGGCUGAGGUCUAUUACAACAUCGACACGAACUGUGUGGAUCCACUUGGCCGCAGUGCACUAUUAAUUGCCAUUGAGAACGAAAACCUGGAGGUGAUGGAGCUGCUUCUGGAUCAUGGAGUAAACACUGGAGACGCUCUGCUCUACGCCAUCCGCAAAGAAGUGGUGGGGGCCGUGGAGCUACUGCUGUCCCAUAGGAAACCCAGUGGAGAGAAACAGGUCCCUUCCUUAAUGAUGGACGCCCAGUUCUCGGAGUUCACGCCUGACAUCACCCCUAUCAUGCUGGCAGCUCACACAAACAACUACGAGAUCAUCAAACUCCUGGUUCAGCGCAAAGUCACCAUCCCUCGGCCGCACCAGAUCCGCUGCGACUGCGUGGAGUGUGUGUCCAGCUCUGAAGUGGACAGUCUGCGUCACUCCCGGUCCCGUCUCAACAUCUACAAAGCGCUUGCCAGCCCGUCCCUCAUCGCCCUCUCCAGCGAGGAUCCCAUCCUGACCGCCUUCAGACUGGGCUGGGAACUCAAGGAGCUCAGCAAAGUGGAGAACGAGUUUCGGCAGGAGUACGAGGAGCUUUCUCAGCAGUGCAAGCUCUUCGCUAAAGACCUGCUGGACCAGGCACGAAGCUCCCGUGAGCUGGAGACCAUUCUCAACCACCGUGACGAUCAGAGUGAGGAGCUGGACCCACGCGAGUGCAGAGACCUGGCCAAACUCAAGCUGGCCAUCAAAUACCACCAGAAAGAGUUUGUGGCACAGCCAAACUGCCAGCAGCUUUUGGCCACACUGUGGUAUGACGGUUUUCCUGGCUGGCGGCGCCGUCAUUGGGCAGUCAAGUUGGUCAUGUGCUUCAUUAUCGGCCUGCUCUUCCCAGUCUUCUCGCUGGUUUACCUGCUGGCUCCCAAGAGCACGCUGGGGCUCUUCAUCAAAAAACCCUUCAUCAAGUUUAUCUGCCACACGGCCUCCUACCUCACCUUCCUCUUCCUCCUCCUCCUGGCCUCACAGCACAUUGCACGCACCAACCUACACAUGCAGGGCCCUCCGCCCACCGUGGUGGAGUGGAUGAUUCUACCUUGGGUGCUGGGCUUUAUAUGGGCUGAGAUUAAGGAGAUGUGGGAUGGAGGUUUUAACGAGUACGUCCAUGACUGGUGGAACCUGAUGGACUUUGCUAUGAAUUCUCUCUAUUUGGCCACCAUUUCUCUAAAGAUCGUGGCUUAUGUCAAGUAUAACAGUUCUCGUCCGCGAGAGGAAUGGGAGAUGUGGCAUCCGACGUUGAUCGCUGAAGCUUUGUUUGCCAUCGCCAACAUUUUCAGCUCUCUUCGGCUCAUCUCUCUGUUCACGGCGAACUCUCACCUGGGCCCGCUGCAGAUCUCUCUGGGCCGCAUGCUGCUGGACAUCCUCAAGUUCCUCUUCAUCUACUGUUUAGUGCUGCUGGCCUUUGCCAACGGACUCAACCAGCUUUACUUUUACUAUGAGACAGAGGCGGCAGAUGAGCCCAACCACUGCAAGGGGAUCCGCUGCGAGAGGCAGAACAAUGCAUUCUCUACGUUAUUUGAAACACUUCAGUCUCUGUUCUGGUCUGUUUUUGGCCUCUUGAAUUUGUAUGUCACCAAUGUAAAGGCUCGGCAUGAGUUCACAGAGUUUGUUGGAGCCACCAUGUUCGGCACCUACAAUGUCAUUUCGCUGGUGGUUCUGCUCAACAUGCUGAUCGCAAUGAUGAACAACUCCUACCAGCUCAUUGCUGACCAUGCUGACAUCGAGUGGAAGUUUGCUCGGACCAAGCUGUGGAUGAGUUAUUUCGAUGAGGGAGGGACGCUCCCCCCACCGUUCAACAUCAUCCCCAGUCCCAAGUCAUUCUGGUACCUGUGCCGAUGGGUUCACGGGCGGCUCUGCGGGUCGGAGACUGCUCCACCUGAAGAAGACACGCAUAAACAUGAAAACCUGAAGGAGUUCACGGAAAAGCAUGCGGACAGGCUUAUACAGAAUCAGCAUUAUCAGGAGGUCAUCCGGAGUCUAGUGAAGAGGUACGUGGCUUCGAUGAUCCGCAGCGCUAAGACGAGCGAAGGGCUGACGGAGGAAAAUUUUAAGGAGCUAAAACAGGACAUUUCCAGCUUCCGCUACGAGGUUUUAGACCUUCUUGGCAACCGAAAGCCUCCUCGGCGGAACUAUUCAUCCAGCAGCGAGGCCACACUUCGAGACGAGGUCAGCGACGACGGCGAAGCCUGUCAUCACAGUGACGGGAGCAGCAUCGGAGCCAAGGGCGUCUCUUUCAAAACCAUCACACCUUCGGAUCCAUCGUACGGGGUGUCUGCCCUCUUCCACUCCAUCUCUGGAAUGGAGGCCGCUGGGACGACGAGAGAGGGUAAACCAAAGAUCAAUGGACUGAGGAAGAACCCCUCGCCCUCCUCUUCCCUCUCAUCAGUGGCUCCAGCGAGAGCUCCAGCACUUUCACGGACUUGGAGGCGAUUUUCCCGCUUCUCGAGCUCCAAGAAGAACUCUUUCAGACGGCUCGGACUGCUGUUUUCUCGCAUGGGCAGCCACCUCCCGCAGCCCACAGGCGUUACCGCUGCAACCUACACUAUCUCAGAUGGUUUGCUUCAUCCGCCCGGGGCUUGGCAUGACUUGGGCUUUACCAGGGGCGACGGCCCUGUCACCAGAAGUGAGACCAACCUUAAUCAGGUGGCAACUUUAAAUGAGGGUGGCAUUGGCAUUGGUGGAAGUGGUGUUGGAAUGUCGACCAAUGGACAGAGAGCUAACGGGAGGGAUGUUUUACUAACAUUAGGACCUCUGUCGCCACCUUCUUCCCUGCACUGUGCUUCCAGUUUCUCCGCCUCCAGCUCCCGCCUCCUGGACUCAAACGAGGACGUGUUUCAGGGAUUGGUCGGGCCCUGCGUUGGACAAAUGCCACACUCCUCCUCCUGCUGGGUUACGGAACAGGACGAUUCGGUAACCACGCAGCUAUAGUAUUGCUAGUCUGUACUGUACAGCACUUUGAUAAUAACAAUAAUCAUCAAUGAGAUCAAUAACUAUUACCUUAACGACAACCGAUCCUUCUGUUUGUUUUGUUUCUUUCGUUUGCUGCUGCAAUAUUUGUCAUUUCUCUCUGAGAUUUAACCAAAUCUAAAGUCAUUUGUGAGACAGAUGGGAUGAGUAAAUGAAACAAUGGACGGCGAUAGAUUCAGAAGGGACAGAAAAGUGCCACGAUCAGCAUAGCAGGAGGACGUAGGCCAGGCUGCAUCUUUGUCUUCAUUACAUAACAUCUUCCAGUACCAGUAGAUUGUUUGUUUCGUUCCAAAACAGGGACAUCAUUUGAUUUGCAUCUUCUUUUUGGUUUGGUUCAAAGAGUACAACUCUCCCCUUAUUAGUGCACCUGCCAUCGGUCAAAAUAGAUUGAAAGUUCACCUCAUUGUGGCUUUAUCUGUAGCUGUUUUAAUCAGGCCACAGAUUAAAAUGGCUCCCUCACUGACCACUACUACUCCUGACUGCCCACAUUUUGUUGGAAUACCACAAAUAGGCUAAUGUAGGGCUUUUCUAGGUCAGCAAUUGCAGGAUGGUGUUAUUAUCAAAACCUGGCAAACAGGUAAGUAAAAGCCAUCAUUUCUGCUCUUACUGAUUAAUUAUGUAUUUAAUUAAAGGGCACCUAUGAUCAAAUCAUCUUUUUUAGGCUGUUUGGACAGAACUGUGUGUAGGUAUAGUCUGUUCACAGUUAUAUUGGGGUGAUAUAAAGACAAUAAGUCUCUUUUUUGAAAUUUCUUGGUGUUAAAAUAAGAUCCAAAUCCCUCGCAUUUUAAGGCCCACCGCAAUGUGAUGUAGAAGUGUGGUUUACCAGCCCACCGAAUUGAUUGACAGCCUCAUAUUAACAUGUCUUCGUAGUAACGCAUACUGUAUAAUCAUAUCAACAAGCCAUGAUGUGCGCAAAGCAACUGGGCUUAAAAGAUCUGUUCAGCGCUCUGUGAUCAUCAAUCAUCAUCAAAUGUGAUCAAAAAUGAGUUUACAAGUUUAAAACGUUUUUAAAGCAGUGCAUGUUUAUAAUAAAGACAGUAAAAUCACUGUAAAUCAUCACCACAGUCAGUACAGUUAUAAAAGAAGACGCUUCAAUCCCGGUUUUUUGGAUGUUAAAUCAGGUUUAUUUUGUACAUGAACUUAAUGGAUGUCCAUAUAGCAGUGGAUAUUACCAUGUAUCCUGUCAUAUUUGCAUGCAAAAACAGUGGCAAGGUAAAUGCGCCGCUGUGUGUGUGAACUUUGUAACAGCAUUGUGUUUGACUCAUCAUUGCAGAAAGGCUUGAAUUAAUGCCACAAGAAAUACAUCAAAUUAAUAAUCCUUGAGAAAUUUCUUACUGUAGUUUUUUUUUUUUUACGUGAGAUGUGCCUUUAUGUUUGUCACUGUGCUGUUUAUCUAAUGCAGCCGAGGUGGAGAUUGAGGCACACUCUGACAGGCACGUGGAAACUGUGGGCAGGGAGAACUACCAUUAAAGGCACAGGCAACAAAAACAGCUAUAAAGUGAUUAAAGCAGACAGUACCAAAUAUUCUGGAAGGUAUAAUAAAUAAUCUAAUGGGUGUUUUGAGCUGAAACUUUACAGACACAUUCUGGAGACACAAAAGACUAAAAUCUAGAAAAGGGGGUAAAAUAGGUGCCCUUUAAAUCCCUAACAUGAUAUAUACCUUAUUAAACAGUUAGAGCAGGAAUUAAGGCUCUGUCAGGACAGUGUAGUUGUAAUGCUUGAGGAAGAGGAAAUGGUUUUCAAUUAAGAUGUGCUAACUUGCAGAAUGAGACUCUCUUUUUAUGCAACACAUUUCUCGUUAUGAAAUAUGAUAUCACUUGGUGGAUUGAGUUGUGAUGCUUUCUAACACCAAUCAAGUUCCUUUUUGGACAAAACUAACCAAACGAAUCAACCAAAAGCACCAGGUUCUGUAACAAACAGUCCAUAUGAGCCAAGUGAGAAAGUGCUCUUAAAGCAGGGGUGUCUAAACUUGGUCCUGGAGCGCCGGUGUCCUGCAAAGUUCAGUUCCAACCUCAAUCAGGCACACCUGGUCUAGCUAAUCAAGCUCUUACUAGGCUUUCUAGAAAUAUCCUGCAGGUGUGUUUAGGCAAGUUGGAGCUAAAAUCUGCAGGACACCGGCCCUCCAGGACCGAGUUAGGACACUCCUGUCUUAAAGGUGCAGUAGGUUAUUGUAUUCAGAAGCAUUUGUUGUUGUGCUGGUUGAAAGUCUCUUCAAAGUCUAAUAGUAAUGAUUAAAGUAAAUGACCUAAAUGUACUUAUUUGUAUUUUUAUAUUUUGGGUAACAAAAACAAAAAUGUUCAUCUAAUUAAAUAGAGUCGGACCGACAUCUCUCAUAAUUCCGAUAAGUAGCCCAAUCUUUCUGUCAGAAAAUGCAGAUUUGAACAACUGCGCAGCCUUUUGUACGAAGCUCCACCGACUGCUGGAGGUUUACGAUCGGUAAAAAUCUGCUAAAUCAAAACUUUUUAAAAACCUAGGUCAAUAUUGGAGUUACCUUUGCACGCUGGAGAAAGGAUGACACAAUGGCUGAAGGAUUUCUCUUAGACAGGUAAUGUUCUGUUUUAAAACUGUUUUAGCUUCACGCAAAGCUGAUGUAACGUUAGAUGUUGUUGUUACAAAUGGGUUAUAUCUUCACAGAAGUGUUGUUCAGCCACUAAAAUCUUCCGAUGAAAGAUUGAUAAGACUAUUUUCAGUUUCAUAAGGGACCUUUUACAGCAUCGAUAAUGUAGAUUUUAAUUAGUUUAACAACAAAACACAAGUAAAUACUGCUAUUCCGCCUAGUCAAGGCGGAGGGACUGUGAUUGAGAGAUAAGCUGUAAGCAUUGUGGAAGAUCACCUACUGCACCUUUAAAGGAAUAGUUCACCCAAAAAUCAUUAUUAACAGACCCUUGUCUCAUAAAGGGUUUUGUUCCACUAACUGAUUUUGCUGUAUUAUUUCAUAAUACAAAAGCAUUUUUAACAAUAUUUGUAACAAAACGGCUUAAUUCUGGCCAAUCUAAAAACGACCUUGUUCUGUUAGAUGUCACUUGGGAAAUAUUUGAAAAAUAAUUGAAGUUUCUAAUAAUUUAGCCUUUGACUUUCUAUAAAGUACAUUUAUUACACUUGUGAUUUAUUAUCUUUGCAUAUAAUUGCAGAAAACUAGUUUAGUGGGGGUGGCACGGUGGCUCAGUGGUUAGAAGUCACCUCGCAGCAAGAAGGUCACUGGUUUGAGGCCUUACUAGGCCACUUGGCGUUUCUGUGAGGAGUUUGCACGUUCCUCCCAUGUUUCUGUGAGUUUCCUCCGGGUGCUCUGGUUUCCCUCACAGUCCAAAGACAUGCGCUAUAGGUGAAUUGGGUUAGCAAAAUGUUCUGUAGUGUAUGAGUGUAAAUGUAAGUGUGUAUGGGUGUUUCCCAGUGAUGGGUUGCAGCUGGAGGGCCUAAAAUAUAUGCUGGAUACCUUGGCGAUUCCUUCCACUGUGGUUACUUCUAAAGGGACUUAGCCGAAAAGAAAAUGAGUCAAUAAAUGUUUUAGUUGUUUAGGGGCGGCAUGGUGGUUCAGCAAUAAGCAGUCGCCUCACAGCAAGAAGGUCGCUGAUUCAGUCCCGGCUGGGUUAGUUGACAUUUCUGUGUGGUCUUUGCAUGUUCUCCCUGUGUUCGUGUGGUUUUCCUCCGGGUGCUCCAGUUUCCCCCACAGUCCAAAGACAUGCAUUAUAGGUGAAUCGGGUACAGAAAUUAGCCUUAGUGUAUCGGAUAAGUGAGUGUGUGUAUGAAUAGAAGAGUGUAUGGGUGUUUCUCAGUACUGGAUUGCAGCUGGAAAAGCAUCCACUGCGUAAAACAUAUGCUGGAAUAGUAGGCGGUUCAUUCCACUGUGGCAACUGCUAAUAAAUAAGGGACUAGGCUGGAGGAAAAUUAAUGAUUGAAUAGUUUAGUGGGUGUUGCUAAUAUAAUGCUUGCUGGCAAAUUUAACACCUUUCUUACUUCUGAACAACAUAAUCUCAAUGUUCAGGAAAAACUUAACCUCUAUUGUGGAUUUUUAUUUAUUAUUUUGCUAUUGUGGUAAAUAGGAAUGCAAAAAACAAAAGUUUUAGAAAUGACCAUUCUAACCUAUUCAGAAGGUAGUGUUUUCCCAGAGAGAUGCAAGGGAUUUUUUCUGUUUACAGGGGCUAGUUGAUGAUGUUUAUUGCCACCUGACUCCAGGAUGUUGGUGUAUUCCUCUCACCGCCUGCGUAAAAUUCCCCAGCCGAAUUACACGACAAACAGCAAGGUCAUGUGGUGAUUUAACAGCCACACGAAUCUGAAAAAGCAGAUCAAUCCAAAAGUCAUUUUGUAAAUCAUUUUUAACAGCUUAUGCUUCUGAUAAAUGUGAAAAGCCGAGAGAUUAUCAGAGAGGAACAGUACAUUUUUGGGUGAACUAUGCUUUUGUUUAAAAUUAACAUGAAGAUGAUUAUUUAUUUCCUUAAUACAUAUUCCUGAUAUGAUUAUGUGUUAUUUAUUAGUGAAUGUUCCAUCAUCUGUCUUCAUCAAGAUUCCUUGUAUUUAAGCUGACUGAUAUUAUUGGACAAUGUCACAUUGAUUGUGCAGGCUAUUAAUAUUAGCCUUUUUACAUCUCAUUAUGAAUGUUAUGUAUAUUUAACAUUCAUUUAAAAGGUUAUAACAAUGAAUUCCAAGAACUGACAAAUUUGCACAUUUUGAAAUGCAAAACUUGGCCUUUAUGUACAAAAAGCACCACCCUGCUCUGACAUUGCACAAAUGUGUCAGUGCCAGCUUUCUAUAUGAAAAUAUCAGUGCUGGACAAUGAUUCAUUUUAAAAAUCGCGAUUAAUUGUACGGUGGUCUAUGAUUAGGCCCACACAGAAUCUGCGCACGCAAAAUUCUGCAGAUUUUCGUCAGAAUUUUCCGCAGAUUUUCGUCAGAAUUUUAGCCCAUCAUUAAUUCUGUUUAUUUACCUGAGUAAAUGUGUGUGAAUCUAUAUUUAUUCAGUUUUUAAGUAAAUUACAGUAAUAUUAUUGACUAAUAUGAAAAUGUUCAUCUGAUUAAUGUACAAUGCAAUUUGUAAAGUAAUAUUUUCUGUCUUUUAUUAGAGAUAUUAUAUGAGAGACUUGCUUUGUUAACCAAAUAAAGUGAAAGUAAUUAGAUUUGCAUUUUAAACAUUAAAUAAAAGUUAAAAAUAUAUAAUUCUUUAUUUCACAUAUUAAGGUUUUUGUUAUGAUACUUUCAAAAUAAUUCUGCAGAAAUCCGCAGAUUUUUACCAAAAUUUACGUUUUUACCAAAAAACGUCUGCAGAUUCCGUCUGGUAUCAUCUAUGAUUAAUCAUAAUUAAUGCAUUGUUGUGCGAAAAAAUCAGUACAGCUUUUAAAAGAAGUGCAUGUAAUGUGCAGUAGGUGAUUGUCAUCAGAAACAUGUUUUGUUGUACCGGUUGAAAGUCUCUUCUCAUUCCACUAGUAAUGAUUAAAGUAAAUGUGUUUAAAUGUAUUUUCAUAUUCUGGGUAAGGCAUAGGACUAAAAAAUGUUCAUUUAAUUAAAAUUUGUCGGGCUGACAAUUUGCAUAAUGCCGCGGUCACAUUAAAGUUUGUGCAUGCGAAAUUCUGUUGUACGACGCUGCGAAAAGGGGCGGGAUUGAACAAGAUGAUUGGACAUUUAAAAAAGCAAACAAUUGGUCCAUGUUUUUAAUUUCUGUCCAUAGAGGUCAUGAUUUGACCUUUGAUUGGUCUCAAUCACGUGAUGUGAUUUCGCAGGUCAGAGUUCACCACGCUUGAACUUUACAAUAAAGCGAACUGCUAAACUUGGCGCACGAGCUUGCGUUUCCGAUCUGCUGCAUUCGCGUGCUUAUGAAUGGAAGUCUGUGGGGGGAUAAGUCUAGUGUGACCGCAGCUUAAUUCUGAUAAGUAGCCCAAACUGUCUGUCAACAAAUGUAGAUUUGUGCAUCUGCCCAGCCGUUCAUGUAGAUCCACCAUUUCCGCAUGUGCCGACAAGAGAGAAAGAUAGAGAGUGACAGCGGUAAAAACAAAUGCUGAAUCAAUGUUGGAGUUACUUUUGCUCACUGGAUGAAGGACGACACGGUGGCCGAAGUGUUUCUUUUAGACAGGUGAUGUUUUUAAAACUAUUUUAGUCCCCCAAAGCUGAUGUAGAUUGUGUUGUUAUGAAUGGGUUAUAUGCACAGAAGUGUUGUUCAGCCACUAAAAUUUUCCGACGAAAGAUUGAUGGGGCUAUUUUCAGUUUCAUAAGGGAUUUUUUUACAUCAUCAAUAAUCAGGGGUGCCGAUGAUGGGGUAGGGAUGUCGCGGAUGAAAGUGAAGGCUAGGGGGCAGACGUGCACAAAUAGGGCUCAACCUUCUCUUGGAUAAAAAAGUGCCCCUCAAAAAUGAAUGACAGUGCCCCCUCAGCAAUCUCAUCCAAACCCCCACACUCCACUCUUCCGGGGUAUAGUGGACGAAAGUGAAGAACGGGGUGGGUGGAGGUGGUCGCAAAUAAAAAUUAAGGGGGGGAGGGUUGUCGUGAAGCCCUAACAAUAUCUCUGGCGGCUCAAAAAGCACGGACCCUUUGAAUCGUCUUAACUUCAUCCCACCCUUUCCUAGCGGCGCCCAUGUCAAUGAUGUCGAUUUUUAUUUAGUUUAACAACAAAACCUAAUUAUAAUAUUAUAAUAUGAUAAUAUAUUAUUAAUGUAUAUAUAUAUAUAUAUAUAUAUAUAUAUAUAUAUAUAUAUAUAUACAUAUAUAUAUAUACAUAUAUAUAUAUAUAUAUAUAUAUACACACACAUUUUUGAACAAUGACAGCCUGUGGCGCGCUCCCUAUAUUGUAUACGGUGCUACUCAGAAUAUUCGCUCUGAAAUAGCAUGUAAGUAGUAAGUAGUAAGCAUGUAGUGAUAAGUGUAAUUUCUGCACCCUGCCGGCCAACCACUAAGCAAACAGUUUUCUAACUAGCGGAUGACAAGAUCUAGUGGGUUGAAUGGCAGGGGAGGGACUGUGUUUUCAACAACAUUAUGCCGGAUACAUCACCUACUGCACCUUUAAGCACUUUUAUUUUAAAAGUACUAAGUAAGUGUAUGAGCAAGAAAAAACGUAUUUUCAUAACAUUUUAGCAGUUAAAAUAGUUUAAAUUUCAACUUAAAUUAAAACACAAACUAAAUUUACAACCAAAUAUAUUUGCUACUGCCAUGACACAAAUCUUACCUAAUUUGUAAUAUAAAUUAAUUUGUGCUCAGAUUUGAUCAUAAUCUUUAUAAUAGGACAAUUGCCGAACAAUAUCAAGUUAGCAUUUAGAAUUAUUCUUUUUAACAGGUAAAAACAAAAACCUUUAAUUUAAUCAGGAAACAUCAUAAACAGUCCAUUCUGUAGCAGCAGUCACCCUAUUCUUUAACAUUCAUUAUUUCAUUUCACUUCAAUAUUUUAAGUUGUGUGUGCAAAUUCAUGUUGAAUUUCACCUUAAACCAAACCAGUUAUUGUUGUUUAACUCAUCGUGCAUCACAAAUGUGGAAAAUAUGAAAGAAAUGCAUCUGAUAUGGAUCUACAGUGUAUGUUUAUGGAACAGAAUAGAUUUGUUUGUUUUUUUCAGUGAGCCGCUGGCACUCACCCGGUGAAGCUUUUUCACUUUAUUGCUAUGAUACGUGCACAUCAUAGUAUGACAUUACUUGUGUUAUGCACCCGGAGGGCUGAAGUUAUGCUAGAAUCUAACAUGACUGAAAUCUUAUUUUUGUAUUUUGAAUAUAGCAUGUACAAAUGAUUUUAAUUUCAAGACUGUUUUAGUCUAGACGAGACCUUAUUUAUACAUAUUAUAACGGUUUUUUGGAGCUGUUUUUUUUUUCUUUGACUGCUUACUUUAUACUACUGUAUGUGAAUAUUAAUUAGUAUAUUAUAAUAAUCAGGUAUGGGGGAGAUGUCCAGGGACAUGCAUCGAAUAAGACAUCCCCCUGCUCUGCGAUGAUGAAUGCUGACGAACCAUGUACAGUGCCUUGCAAAAAUAUUCAGACAUUUAACCAACUCUGAUAUUAUAUUCAAUUCUAGAUAUGGCAUUGGAGAUGCUGUUAUUUCAAUUUGGCGUUGCUUUAUGUUAAAGAAAUAUGAAUAAAAUGUGGUAUAUAUUGCAUCAUAUGAUAAUUAUUGGUAAGUAUUAAUAUACAGUGCAUCCGGAAAGUACUCUUAGCGCUUCACUUUUUCCACAUGUUUUAUGUUACAGCCUUAUUCCAUAAUGGAUUCAAUUGAUUUAUUAGCUCAAAAUUCUACACACAAUACCCCAUAAUGACACUGUGAAAAAAAGAUUUUUGGGAAUUGUUGCAAAUUUAGGAAAAAUAAAAAAGCUGAAAAAUCACAUGCACAUAUGUAUUCACAGCCUUUGCUCAAUACUUUGUUGAUGCACCUUUGGCAGCAAUUACAGCCUUAAGUCUUUUAAAAUAAGAUGCCACAAGCUUGGCACACCUGUCUUUGGAAAUUUUUGCCCAUUUCUCUUUGCAGUACCUCUCAAGCUCUAUCAGGUUGGAUGGGAAGCAAAGGUGUACAGCCAUUUUCAGAUCUCUCCAGAGAUGUUCAAUAGGAUUUAGUUCUGGGCUCUGGCUGGGCCACUCAAUAACGUUCGCCGAGUUGUUGUGAAGCCACUCCAUUGAUAUUUUGGCGGUGUGCUUUGGGUCAUUGUCCUGCUGGAAGAUGAACCGUCGCCCCAGUCUGAGGUCAAAAGCACUCUGAAGCAGGUUUUCAUUCAGGAUGUCUCUGUACAUUGCUGCAUUCAUCUUUCCCUCUAUCCUGACUAGUCUUCCAGUUCCUGCUGCUGAAAAACAUCCCCACAGCAUGAUGCUGCCACCACCAUGCUUCACUGUAGGGAUGGUAUUAGCCUGGUGAUGAGCGGUGCCUGGUUCUGUCUAAAUGUAACUCCUGGCAUUUAUUCCAGAGAGUUCAAUUUUAGUCUCAUCAAACCAGAGAAUUUUGUUUCUUAUGAUCUGAGAGUCCUUCAGAUGCCUUUUGGCAAAUUCCAGGCGGGGACUGGCUUCCAUUUGGCCACUCUACCAUACAAUCCUGAUAUGUGGAUUGCAGCACAGAUGGUUGUCCUUCUGUAAGGUUCUCCUCUCUCCACAGAGGAAUGCAGGAGCUCAGACAGAGUGAUCAUCGGGUUAUUGAUCACCUCCCUGACUGAGGCUCUUCUCUCCCAUUUUCUCAGCUUAAAUGGCCGACCAGCUCUAGGAAUAGUCCCGGUGGUUCCAAUUAUGGAUAAUGGAGGUCACUGUGCUCAUUGGAACUUUUAGAGCAGGAGGAAUUUUUCUGUGACCCUCCCCAGCCUUGUUUCCUCGAGACAAUCCUGUCUUGGAAAUCUACAGACAAUUCCUUUGUCUUCAUGCUUAGUUUGUGCUUUGACAUGCACUGUCAACCCUGGGAUCUUAUAUAGAAAGGUGUAUGCUUUUUCAUAUCAAGUCCAAUCAACUGGAUUUACCACAGGUGAAUUCCAAUUAAGCUGCUGAAACAUCUCAAGAAUGAUCAGUGGAAACAGAAUGUAGCUGAGCUCAAUUUAGAGCUUCACAGCAAAGGCUGUUGAUACUGAUGUACAUGUGAUUUUUCAGGUUUUUUAUUUUUCAUAAAUUUGCAACAAUUUAAGAAAAAACAUUUUUUAAACAUUUAUUUGUUAUUAAGGGGUAUUGUGUGUAGAAUUUUCAGGAAACAAAUGAAUUUGAACCAUUUUGGAAUAAGGCUGUAACAUAAAAAAAAUAUGGAAAAAGUGAAGCGCUAUGAAUAAUUUUCGGAUGUGCUGCAAAUCCAACAAAAAUAGCAGUAUUUGAGAAAUAUUAAAAUGUGCGGAAUGUCAAAACAAUUUUGUUUUAAUAAAUUAGAUUCGGGGCUUUGAAAUGCAACAUAACAGAACACAAACUGCAGUGUGGGACUGUGUAAUCCAGUAACAUGACAGAAUUGGUCAAGGCCUGAUUACUUUUGGCAAGGCGCUGUAAAUGCUUAUGUAAACACUUGAAUGAAAUACAUGCUCCUCUUGUAUGAUUCUUACUAGUUUCUACUUUUCCUUGGAAAUGUGUGAACUUACGACGAAGAUUUAAAAAAAAGUAGUUUCGACAGCCAGAAACUGAACUCAAGGGAAUGCCGAAUAUGCAGAUUCAAUAAAGCUAAAUAACCCUGCUGUU |
| Medaka | *sost* |  |  |
|  | *fgf23* | XM_020714269.2 | GAGCCTTTGGCCCGCCCAGCATATTTTTACAAATATGATCTTTTTCAAACAGCATTTTTTCATCTGCTCCTGATTCACAACGATTTGAATAAACACATACTAAGAAATGAAACTTTGAGCTTAACTTTCTTTATATACGTCCTCCACGTTATAAACACTGTTTTTATCTGAGTGGGTCTTTAATATAAATACTTGTATTTCCCTCATAAAGAATAATAGAGGTTAGTATCTCTTCACAATCAATTATCTCATTTGCTTAAACATGCCTCCTACTCCTCAGTTCCTCAGAGCACACCATTTAATCAGACTGTTTCTGTTGTGTTGTTTTTTTTTAAAATCAAACACTATCATAGCAGTTACTTCAAAAGACAAGATCATATTTTTAGGCATCATTATTTTTTAGCTCATTACACAATTTAAAGAGTAAACACTTTCCACTATAGCCGTGTATTTGTCACCTTTTAAAAATAATTTATCGATACATGCACAAACGAACACACAAAACTGTAATCTGCATGTAATATGTTAGAAGTTCCAAGAAACTGATAAAAAAAATGACTTTTCTTTAGCCTGTAATAGGAAAACTCAGGCATGATTTTTAGTATTTTTTAGCTTTGTGTCAATTTCTGGATTTCAAAAAAAGACAGTTTTAAAAAATGAACTTCAGATGGATGGATGGATGCTTGACTAATAATATATTTGAATACATAAAAAGCGCATTATTCAGTAACTAAAAACAGGAAATTAAAATTAGGTTTTTGCTCGATAAAAGTGCATTTGTTGTTCAAAAAAAAGCAGGATACTTAATTAATGAGGTTCGTTACTGTTTTTCACTTTGTGTTAAATTGCGGATTTCAAAAACGACAATTTAAAAAAAACTTGCAAAAAGATTTTCTTGCGATTTCCCCCTCTTGTTTTAAACACTTGCATACAGCACACAGCATTTTTTAAAATCCAGAATACTAAAGGAGGCAAACATCATATTTGGAGGCTAAAGGTAACGTTGCACTAAGTTGAAGAATGCTGTGTGAGGAAGTGTCTCCATCTGCTGGTGAAACGGTGGATCTCTTCGGTTTGCAGGTGCATAAATTTATCAAACTGAACCAAATTTATGAATGGAAAAGTTATTAAAAACCGAGATTTAACTACTAAATCCAAATATAAACATATGCAAATTAGTCAACCCTGCAGTTTGAATTATATTTAAATTATATTGACCCGATTGACATAAAAACACAGTTTGAGTTTAGTATAAGCTAT  TAAATATTCATATTTCTGTCATCTTTTTACTTTAGCCAAATATGTTTTTCCTTCCCAAACTAAAACCAGAACCAGAAAATGAACAGTTTAAGGTGGCTGACTCAAGAAGTGAGCCCAAAAATAGGAGTCGTGTTGTCTGACCAACAACATTGGGACACAAACAGAGGTGGAAGTGGCCCTTCCGCTGCAGCAACCCGCACATCGGCTTTGGGGGCGAGTTGCTTTTTTTTTTTTAACCCAAAGGATGTGAGCAGGAGTGGCACTCAGGGCCTTTAAATACGAACTGTCACCAAACTTCGGCACAACGGGCACCCAACAGCTCGCATGGGACAGGTGATGAGGGACACTACCATGCTGGCGCTCGTACTCGCCGTTCTACAGGGGTTTTCTGTCCGGCAAGTAACCCCAGATCCATCCCCGCTCGTTAGCTCCAACTGGGGAAACCCGAGGAGATTCATTCACCTGCAGACATCCACGGATGTCTGCAACTTCTACTTGGAGATCAGATCAGATGGAACCGTGCGCAAAGCTGCGAGCAGAACATCATACAGTGAGATGGG  CTTTTATTCTAGTATCTTCTTAAATGCTGTGCACTGGTCTTCATTTGGATTTATCTGGAATAACTTGGCGCTAAATCTTTTTTATTCAATCTTTCCAGGTGTGATTTTACTTAAGTCTGAAACGAGGGATCGAGTCGCCATCCTTGGAGTCAAGAGTAACCGTTACCTGTGTATGGATCTGGAGGGTAATCCATTCAGCTCGGTGAGCATACAGTTCATCCTTTTCATTTTATTAGTCAATGCCGACTAAATCTTGATCTTTGATCGTCTTTTCTTTTATTATTATTTAACTCCATGAAGTTTTACGCACCCCCTCACACAATTCTTCTCAACAGGGCCTGTAGACTTAAAAGATTGATCCAAAATAGGTATTATTGATCATCCGAGCAGCTTCTTCAGAACATGTCTGAACTGTTAAACCGTGCGTGAAAACGCACTATTTTTACGCACAAACGCAGCATCGGGTCCTTTAAAAAATCCGGTTTAATTGTCTGACTACCTGCTTTCTGCCTTAAAGTAGTTAATATAACGATTTGGTCGGATCTCTGCCACATTTGCAGAGTTTATGAACTTAAAAATCACAAAAAGTCTGTTTGTTTAAAATACCCGACGCAAAAAAAAACATGAAAA  ATGTTTTGAATGCACCAAATAAGACGAATACAAACAATTTAAATAACTTTACATTTTTGTTTAAAGTTTTTTTTTTTTAACCAAAAACAGTTTTTCCATGCGTAAAATTCTTTTCATGAATGGAAAATGTACTATAGATAAACATGAATTTATTTTTTAATTCGAATGCACTGAATCCAATGAAATTCCTCTCCGTTCCCCCCTTTTTTAGCCTATTTGCCTCAGAGAGGAGTGCCTCTTCAATCACAAACTCCTGGAAAACAACCGAGACGTGTACTACTCCACTCGGACCAGCATCTTGTTCAACCTGGAGGGGTCCAAACAGGTGTACATCGCAGGTCAGAACGTGCCGCAGACCTCCCTGTTCCTACCCAAGAAGAACACGGTGCCGCTGGAGCGCCTCATCCCGCACAGGGAGAAGAGGAAACGGATCGTGGATCCCTCGGACCCGCACGGAGTCTACCACAGACCGGCGGAGGAGGGCTCCGACTCTCGGGCTGTGGCGGAGCAGGACGACGCCGACGTGGAGGTGGAGACAGAGATGGAGATGGAGGTCGGTG  ATGACGGACGCAACTTATCCCGGGAGAGACCGCTGGTTCCGTTCAATGACGACCCCTGGAACGTGAATTCGTTCAGACAGTCCAGCCCCCGAGGCACCGGAACAATGGGCTAAGCCGGTGAAACATTCAAATATAAAGCAAGCTTTATTTTGAAAAACTGGCATGAGAGTAACACTTCTGTTGCTTTGCTTTCACACTGACTTTGCATAGTTTAGTTAGTGCTAAATCTTTCTGCAGTGTGTTTTTACACTGAATGAACATTTTTAGACTTGACTTGATACCTTTTGCACAATTCTTGAAAACTCTTATTTCCAAAAACTTAATTCCTTTTTACAGAGCCCTGGACAGGACTTTACAAAAAAAAAAGACAAAGAAAAAATTTGCATTTCTTCTGGCCCCCATTAGTATTTTGTAAGCATATGTTAGTTTTTTTTTGGCCACAAATTAGTATTTAGGGGTTTCAAATGAGAAATAAAGATACTAAAATACTATTTTGTGTGCCCAAATTACTTATTTGTGCACAGAAATTAGCATTTTGTGCAAGGAAATTAGTATCTGTGGGTGCAAATTAGTGGTAGGAATGGAAGATACTTAAAATGCTTATTTGTGCCCATAAATGTACACACAAAATGCCAATATGAGTCCTCAAAATGCTAATAAAATGATACTTTTGTCATGTCCAGGGCTCCTTACCTTCCCATCTTCTTCTAGTTTCCACAACTTCCTAAGAATTTCTGCTCTTTAAATGTCAAGATGGTTAAGATACATTTATTTTCCCCCCACAAATTTAGCTGATATCCTCTGCAACTCAACCCATATTTTTATGAATTTGTGTTATATCCTGTTTATTTACATTGTTCAAATGTTTTATACAATCTTAGGAACCCATATTCATGTCGTCTAATGACAAACCAAATTTAGTCTACCTCTGGAGGACTGAAGGGTTTGACCGCTGAACTGAAACCAGCAGGCATGAACAAATATGAGACTATTAAAAAATGCAATAGGATTAGCCTTCATTTTAGCCTTCTTTAAGTATTTCAGCAGTTTGCTTTATTAATTATGAAGAAAGTCGTCTTATAACAACATCTGAATTTTCCCACCAGTAGGATGGAACTCATAACTTGAAAGAAAAGGACAAACAGGGTCAAAAAATAAATGTTAATTTTGTAGGTGACTGAGTTGTTCTCATCAACAGCAAACACACAGCAGTCCCTAAAAGTATTTATTTATTGAAATATTTTCCTTTCACTGTTTATTTATAGTATTTATGGTAATGTTGGAGTTGTACATACAGAAAAAAAAGATTTGCTTTGAATGTATGAGCTGGAATTTAATTTAGAAAGAGTTTTGCTTGAATTTGGGACCAAATAAAGTTTAAAAAAGTC |
|  | *Col1a1a* | NM_001122918.2 | GCAGGCAGTAGGGTGTTTTCTGCTGGGGTCAGGAGGACTTTGGACAGACGAGCGGATCCTGCCCCAGACACCCCTGTATGAGTGGAGTGTAGCAGAAGGAGAAAACATCCCCAACTTTCTTCCTCTCTTGCCTCAAAGAGTCTACATGTCAGGTGGCATCTAGACATGTTCAGCTTTGTGGATTTGCGGCTGCTGGTGCTGCTCAGCTCTGCGGCACTCCTGATCTCAGCGCAGGGCGAAGATGACCGTAAGTCTCATCAGTGTTTCTTGGAGCTTCTCAGACCCTTTCTCTTTGACCGCGGGACTCTGTTTTGGGCCGGACGCCGGTGAGATTTACTGGAAAAAGGCAAAACTTTGGGGGCTCGTGCTGCTTTGCCTCCAGACGGGCAGACAGATGTGCGTTTTTTTTTTACAGCGCAGGGTAACACATGAACCCCCCGCCACCCCCACCCTTGCAGAAATGCACCAGAACAAGTTGCTGCGTGCTTTC  AACTCTTTCTTCCAGGCAAGGCTTTCCTACTAGAGACAAGTCACAAGGAAAGCATGATGCCCAACTTTTTTCATGCCTCCAAAAGTAGAGTAGTAAAATAACCTTTTTATGCTTTCACATGGATTCCATCATACATCATTCCACAAATCCTTGTGCCTCAGCTGGCCTACCACCCCTACAGGTACGAGTCTGAGTGATGTGGCAGTAATGAGCTTGCCTAAGGCCAAGGATTGCTAGACAGATGAGATCCATGGTGAGAAAACACAGAGGGGAGAGCAGGCACAGTCTTGCCTGCAGGACTCATGAAATGCTTGAACCCGCTTTAGTTTAACATCTTCAAGCGTGTAGATCAGCTCTAGCAGGCCAAAGCAGATCGGGCAGATGGAAAAAGCTTTCTTCCTGTGAGAAGACAAAGGAAAC  GAGAACCATACAGACTAAGTCAGCTTTTCCAGATGTTCCTACCATCTGGAACAGAATCAGGAGTCTAGAGATGGGCCACGACCCTTACAACAGTGTCTGAGCGACACCTAGAGGCCCCTTCTGCACCTGCACTAGCCACATCCCCACGCTCTTCTTTTTTTTTCCCTCTCCTCCTTTTCACTTTCTTTCCACTCGTTTTTGTTTCCTGTAATTGCAAACACATGTGCTGTGTTTCCATGGGCTGCTGCCAAGCTGACGGGCCGCCGCTGCTTTTCCTCTCTGAGTTCAGCACTTTGGCATGCTGGAAGATTTACAAGCACTCTGAAAACAGGCCTGGCCAGAGGCTGGCGTCTGCAGAGGGGTGCGCAGCGGCACCCATGCAGGTCAGAGAGGAGGACAGGACCCTGCTCTGTACCAAAACCATCTGGAGACATCTGCCTTTTCCTTGGCTGGTGTTTTTCCACTTACAGCAGAGGCTGACCTGCGTCAG  GCCCAGCAGGATGCTGACCCCACAGTCACCTCTGACCCTTTCAGTTTAAGTCCCTTTTGTCTAAGTTTGATCCTCTTTGCAGCCGTGCATCCACAAACAGAGGATTTGTCAATCTATTTCTTCCTTTTAAAGTTGAGGACAAACTAAACGTAGTGGTGCAGAAATTTGTGACAATCTTTGTTCTGATCGTTTTCTGCCAAATATGACTCAGCAAATTAGGAAAATATAGCAAATCTCTTCTGAAAATGGCTTTTGCTCCCCTTAGTTTAGTCGTACATGAGTGTGTTTGGGAGTGTAGCTCCCTGACCTGGCTTGTGTTGCCCTCTGCAGGCACAACCAAGACCTGCATCTATGAGGGTCAGGTGTTTGCCGACCGGGACGTGTGGAAGCCCATGCCATGCAAGATCTGCGTGTGCGATGCCGGTTCCATCCTCTGCGATGAAGTGGTGTGTGAAGAAGTGACCTGCGCCAATCCCATCATTCCCCACGACGAGTGCTGCCCAGTCTGCCCTGACGACGGTATGAGCCTTTCTTCAGAACACATAAAGGCCGCAGTGCAC  AGTCAACCTAAGCTGTTTGACATCTCCAAAAAATCCAAACCTCTCACACTCCCACCTTCACAAAGGTCATCTTCAGGATTTACCTGAGGGCCTTGAGCTCCATGTTTTTTGCTCCACATGATCTTTTCAGAGCTTTCAGTTTTGTCTCCTCCTTTCCTGAGAAATATGGAAGGACACATTCATGCAGTACATTCCTAACCTCCTCGCCCTTATTTCTCCTCTTCAGGCTACAATGAGGGCGCTGTGGAGGTATGTAGCATTATTTTTTTCACCCATCTATACTTTTGTTATGGCTCTCTCAGTTGTGTCAGTTAATAGAGCCACTCAATGATTATGGTGCTTCGTATAAAACAAGCAATCATCTTTTGCACGATTAGTGGTGTCATGATGAAGCTGATATTCGTTCTTTTAACCACAGGGAGCCAAAGGTCCACGTGGGCCCAAAGGAGAACGGGTAGGTCCAGCTCTTTCCTTCTTTGGATTTGATTGGATCTGAGCCGATCCTGCAGATCTTTTGCTGCATTTGAGGCAGCAGAATGCGTCCAGGCTGAAGTAAAAAC  AGGCCTCAGATGTGAGGACAGGGTCAATTCCAGGGCGGCTTCCACATTCGGCTTGACGTTGCTCTCTCTATTTGTTGTTCTTTGCGAACATCAGAACATTTCAGGAGTGAAAAGTTTCATAAAAGCTGTTGTTCTTCTGCATTAATGCATCGTGTTTGGACGACAGCATTCTGGGAAACAAGGAGCCTGAAGGCATCAAAGGAAACCCAGCAAAGTTCAGTCTGGGTCACAGCAGCAGAGACTTTGATTCATTTCAAACTTTCTTTAAACAATGTTATTATTTTTAGTTGAACTGGTGCTATCTGAGATTAAACAAGGATTAACTGGATCATGCCTCAATCAAATGCAAATACTTAATCTGATATTGGAAATAATCAGTCTGATTATTTGTAGATGGAATTTTTGATAAATATGAGATGTTATGGAGTAATTCTGCCACAGACAGAGGCTGTTTGTGTCAATCCTTACATCAACATCCCGTGTCACGTGGCGACTGCCAATCCATTTTTGGTTGGTCACAAGATCATTTTGGCTGATGGATATGCTAATCCATATGACTG  TGGATGCGTCTGTAAAGAGAGAGAAAAAACATTTTAGAGTTGCAGACCATCATGTGACTCAGTCATGTGACACAGTGGTCAGGTGTTTAGAGCCCCCCCCCCCCCCCCCTCTCTTTACCACATGGTCTTACTTCTGCTTCCTTTTTTCTCAGGGTCCTGCTGGTCCACCTGGCAGAGATGGTCAACCTGGCCGACCTGGTCTCCCTGGACCTCCTGGCCCCCCTGGACCCCCTGGCCTTGGCGGAGTAAGCCCTCATTTTTTGTGTTTTATGTGAATTAAGTATCTGGCTGCTTTGTATACAGTCAGAGCTGAAGGCCATAGTCTTAGGGCTCAACTTTCCAAAACTACCACACAGCATTTTTGGTAGCTCTTCTGTACAGGACAACATTCATAAACAGCATTACTTTGAGCTCTGGTGCATTTGAGGAACCCTGTAAATAATGGCTCTTCTTACTCTAGTCTGAGATACTTAGAAAACTGAGGTCAAGAACAATTCTTCCTTGATAATGACCTTGAGTCTGGAACAGAACGTTGGATTTTGATGCATGGTCAGTTCACT  CCTGGGCTCCTGCTTCTTAACATTTGGAGACAACAGTTGCTGAGCCAAGGCTCTACAGTTGGTTCTCTTTGAGGAACAATAGTCAAACTCTGCTGAACCAAATCTTTAAGACAGACATAGCTGGGATTTGGCTTTACAGTTCCTCCAATGTAAGACTCTCACATGACCCATCATCCTGAACAATACTAACTAACTGAAGTAGATATTTACCTTCATTACTGCTATTCATATGTTACACACAGCTAATTTTATGATACTCCATCAGTGTGGAACTGTTCTCCAACTATCCATTCTGGGTCTTTACTATAAGGCATGACACTCCCACAGGCATTTTCTTGTGGCACAATTCAAACTCCTGCACATTTAGACGATGTTTGATCTTCTTTGATGTTTTGAAGAAGAAAAAAACTGGATTTGCAAAGGAAGGATTATGATCATGTTGCAGAAATAAAATGTATCATTTATGGTGTCTTCTGGTTTTTAACAACATTGCTTTGTTTTTCCAGAACTTCTCCCCACAGATGTCTGGTGGTTATGATGACAAATCTCCAGCTATGCCTGTCCCTGGACCCATGGTGCGGCACTTTGACACACAGACACTATTTAATAGTCTTTTTGTAATTGGTGATG  ACACAATTCTGATCTCTGCTTGTCTGGCAGGGCCCAATGGGATCTCGTGGTCCTCCAGGACCTCCTGGTGCTAGCGTGAGTGCAACTCTCCTCTGCTTCCTTCACTGAGTACACTTAAGATTTAAAAAAGAACATTTCACAACTTCATACCATACCAAACCGTTCATTACCATACCTAACCACACCAAATAACGTGTCATTGGCTACGAGGTTGGTAGTTTTCTCACAGCCAAAAGGCTGGTGGTTCAATTTCCTGCCTACCCCAGTCACATGTTAAAGTGTCCTTGATAAGGCACTGAACCCCCATTTGCCTCAAGGGGGCTGTAGAGCCTCTTGCCACTTCTATGTGAATGTGTGCATGTTAGAGTGGGTGAATGGGTAGGTGGGAGGCAAAGGAAAAGAGCTTTGAATAACAACTGTTAGAAAAGCTCCACATAAGTATGGGCTATATAACATATCATACCACACATTATTATATGGTATTACCGTACCATACTGTACTATAATATACTGCACTGTACTATACTGTAAATGGTAAAAGGCGTATACATGTATAGCGT  TTTTCTTTCGTCAAAGGCCCAACATGCTAACAGTCACACACACATTCACACACAGGCGCCAACCTTCCACCAGAGGCAAGGTTAGGTTCCTTGTCTGTTGTCCAAGTATACTTTGCCACATGTACTATACTATACTATACTATACTATACTATACTATACTATACTATACTATACTATACTATACTATACTATACTATACTATACTATACTATACTATACTATACTATACTATATCATACTATACCTTACCATACTATACTGTACTATAATATAGTATACTATACTATACTATACTATATTATATCATATCATATTATACCTTACTATACTATACTGTGCTATATTATACTATACTGTACTATATGATACTATACTGTACTATACTATACTATACTATACTATACTATACTATACTATACTATACTATACTATACTATACTATACTATACTATACTATACTAGACTACACCATACTGCACTATACTTTAGTAAATATGTCACTACACTAAACTATATTATGGTAAAAGGGACACTGTACTATACTACAGGGTTCCATTCCGTAGTGTGTCCATGCCGGATTGTCAAAGTGGAACATTTTGCATGATTTCTGCATTTCCTTATTCCAACATACCTCCCCTATAGGATGGTCATTTTCATGTCACTGCGTGCTTCGTGTTTAGGCTGGGTAACAGTGAAAAC  CTGCACATACTGTATTCTGAGAAACACTACTGTAGTACTTTCATCTGTACTTAGTCAAGCTGAGAAAGATGTGAATATCTAACCAAACAACTGAAAACAATTTACTTAAACAACATAAACCTTTAGGACCGGCATCAGAAATGAAAAATAAACAAACAAACAAAAAGATTAATTCTGAAACAACTAAATGTGCCGTTTGATTCTATCTTAATTACATTTTTTTGCTATTTTATTGTCATTTTACTTTCAATGTTGAGTTTTAGCAAACTCAAACAGCACCCCTGTATGATCACGTTTTGCCATGAGCTGTGCACATTTTAAAGGCTAACACATTCATTTTTTGTGTATCTAAAAAAACAGATTGCTTAGAATCAAGAAATACTGTACTATGAAAGCCTTTGATTAATCCTTAAAGCATAATATCGAAATCAAGATTGTTGAAACATCACATAAGACCAGACCGAATGAGAATGGCATTTAAGTCAGCATGACTTACATAAAAAGTTCTTAAAGTGTAGAGCTGTACTCAGCTGTGCCGTCATGAGAAGTTTAGGTTACTG  TTACCTTAAGTGAAAAACTGTCAGGCATCAGCAAAAACGGACTATTCCTTAGTAAAGGAGAACAATGTCCAAATGTAAGTCATAGAAAAATGTGGGCTTACTTGTGGAACTGTTTGTGCCTACAGGGACCTCAGGGATTCACAGGTCCCCCTGGTGAGCCTGGAGAGGCTGGAGCTCCTGTAAGTCTTGAATCAATGTTACAGAGTTCATCCTTTCATTACAGGTTTACCTTAAAAGACCTTCCTAACATTGTGTATTTTTCATCTAGGGTCCCATGGGTCCACGTGGACCTGCTGGTCCTCCTGGAAAGAATGGAGAAGACGTAAGTAGTCCAAATCGCCTAACATTTAGAAATCTGCATATGGACCTCAGTTCAGATTCAAAATGTCCGTTAACTCTTTCTCCTCCCATTTACTTGATCAGGGTGAATCTGGCAAACCCGGTCGTGCTGGTGAACGTGGACCTCCTGGCCCACAGGTAGATTGTGTAC  AAGTCTCTCACTGGGACAGCTGATCTCGAGTGAAACAGAGTTGATGTGCTAACATTGTTTGACCTCACCAGGGGGCTCGTGGTTTCCCAGGAACCCCTGGACTGCCTGGCATCAAGGGACACAGAGTGAGTACGAGGCTCCATCATGGCTCCACATAACAGCTGGAACTGAACATCTCACGTCAGCCGTTGTAACGTTCGCTCATCTCCTGTGTGTCGTAGGGTTTCAGTGGTCTGGATGGTGCAAAGGGAGACACCGGCCCTGCAGGACCCAAGGTAAGCGCCCCTACTTAACAGTAACTCACATGTGACCAATCGAACAGCTGGCTTTTTCTTTTGCCGCTGTGTTCTAGCTTATGCGATGTGCTTTTATCTACCTTTGTATTTTTCCACTTCCCAGGTCGTCATTGTAAATAAGACCATGTTCTTCATGGCTTACCTGGTTAAATAAAGGTTAAAATAATTATAGTAAACAAGGAGAAATGTGTGTT  AAGGTTCAGCCTGCTATTATGAGGTCCCTGGGTTATGGTGTCAGAAAATATCCTTGATGTTCAAATTATAACAATCCTAGTGCTGGACATCAAATGTTAGATTTTCTGTTATTGGCCTTTAAAAACTTTGATTTAATCCAGTCTTACTATAGCTGCCCCCAGATGACCTCAGTTCTGAGTGGAGCCATAGAGCAGGAAAACCCATGCTCAAGTGCTTCTGCAAGCTTTCTTCCACAGTCACTGCTGGACCTGTCCAGAAGCTGCTCTAAACAAACATTTAAGCTAGTCAAAGATGCAAAAATCACGACCTATTTTGATGTGATAAGCTGGCCTTTGTGTTTTATTTTAGGGTGAGGCTGGAACTCCTGGAGAGAATGGAACTCCTGGTGCCATGGTGAGGACACGAGCACATCCCAACACCACAGTCCTCACTGAGACCTCACCCCTAACCCTGACTGTGTTTGCTCTTCCAGGGACCTCGTGGTCTGCC  TGGCGAGAGAGGTCGCGCCGGTCCTTCTGGAGCTGCTGTAAGACCTGCGGCACCAACACTGCCTGAGCACTGTCCTGGATTAATATCTGTGAATGTACGGTAACCTCTGCGCTTTTCTGCAGGGAGCTCGTGGTAACGATGGAGCTGCTGGTGCTGCTGGACCTCCCGTAAGTCTGTCCTTCTCTATAACTAACAGAAACTCACCAAAGTGTAGATGTTTATCTTCAGGAACTTAGAATAGAAAATAGCCTAATAAAATGGATGATAGAAACCACTCAAAAAAATCCCTTTTCTTTGATAGCAGTGTTACCATAGCAACCAATAGAAGGCGGTCCTCTAAAGAACGTTTTTACGTTTTGTACTGCTACCACAGCTTCTACTTAGCTGGCTAAATTAGCAGTTTCTGAGCCACAATAGTCTATCTGAAGCACTTGACCACATCAGCCATGTCAGCACGACATCTAAGGAACTGTTTCTGTTTGTCTGAATCCCTTTTGTTTGTGCTCAGGGTCCCACTGGCCCTGCTGGACCTCCTGGATTCCCTGGTGGCCCCGGAGCAA  AGGTATGTTGATATTCAAGATAACTATTATTGAAAAAGGAAAAGCTTCCTTTTAACGTGTTTACTTCTGC |
|  | *TRPC5A* | XM_023948991.2 | AACCAACACT GAGCCCTCCT CATCCTCCCA TTCACTCCCC GAGGAGCAGC CGGAGCCGGCGGCATCATGA TGGGGCGCGC GAGGACGCAG CAGCGACATC ACGGGCGTCA CGAGCGATTC CGTCCAGGTG GCGCGAGGAG AACAAGCTGA TCCCGAGGAG GAGGAGGAGG AGGAGAAGGA GCCCGGGAGA AACGGGGGAG GAAATCCCGC GAGAGAGGAA AGAGACCGAC AGGCAGAGAC CGACAGAGGA CCTGCAGCAC GTGCGTGCGG GCGCAGGAGC CCAGAGGAGC AGGATAATCC TAATTTTGGG AGATTTTTCT TTTCTTTTCT GACGGAGAAG GAGGCTCTCC GCTTTCAGGT CGTCCAGATG AATCCCATGA CACAGCTGTA CTACAAGAAG GCGACGTACT CGCCGTACCG CGACCGCATC CCGCTGCAGA TCGTGCGGGC GGAGGTGGAG CTTTCTGCAG AGGAGCGGGC CTACCTCACT GCAGTCGAAA AAGGTGACTA TGCCGGGGTG AAGCACGCCC TCCGCGAGGC CGAGGTCTAC TACAACATGG ACGTGAACTG TCTGGACCCG CTGGGTCGCA GCGGGCTCCT CATCGCCATC GAGAACGAGA ACCTGGAGAT CATGGAGCTC CTGCTGGACC ACGGCATCCA CACGGGCGAC GCCCTGCTGT ACGCCATCAG GAAGGAGGTG GTGGGCGCCG TGGAGCTGCT GCTGTCACAC AGGAGGCCCA GCGGGGAGAA GCAGGTCCCC TCCCTGATGA TGGACAGTCA GTTTTCAGAG TTCACGCCGG ACATCACTCC CATCATGCTT GCTGCTCACA CCAACAATTA CGAGATCATC AAGCTGCTGG TCCAGAGAAA GGTCACCATC CCGAGGCCTC ACCAAATACG ATGCGACTGT GTCGAGTGTG TGTCCAGTTC AGAGGTCGAC AGCCTGCGTC ATTCCCGCTC CCGACTCAAC ATCTACAAGG CTCTGGCCTC GCCGTCCCTC ATCGCGCTGT CCAGTGAAGA CCCCATCCTC ACCGCCTTCA GACTGGGGTG GGAGCTCAAA GAGCTCAGCA AGGTGGAAAA CGAGTTTCGT CAGGAGTACG AGGAGCTTTC TCAGCAGTGC AAACGTTTCG CCAAGGACCT TCTGGATCAG GCCAGAAGTT CAAGAGAGCT GGAGACCATC CTGAACCACA GGGACAGTGA CCAGAGCGAG GAGCTGGACC CCCGGCAGTG CCACGACCTG GCCAAGCTCA AGCTGGCCAT CAAGTATCAC CAAAAAGAGT TTGTGGCGCA GCCGAACTGC CAGCAGCTGC TGGCCACGCT GTGGTACGAC GGCTTCCCCG GCTGGAGGCG GCACCACTGG GCCGTCAAAC TGGTCACATG TTTCATCATC GGACTGTTGT TTCCTGTTTT CTCCATGATC TACCTGTTGG CTCCAAAGAG CGCGCTGGGG CGATTCATTA AGAAGCCCTT CATCAAGTTC ATCTGTCACA CAGCGTCCTA CCTGACCTTC CTCTTCCTCC TGCTGCTGGC CTCGCAGCAC ATTGCCCGCA CCAACCTGCA CATGCAGGGG CCCCCGCCCA CTGTTGTGGA GTGGATGATC CTTCCGTGGG TGCTGGGCTT CATCTGGGCC GAGATTAAAG AGATGTGGGA CGGCGGATTC ACGGAATACA TCCACGACTG GTGGAACCTG ATGGACTTTG CCATGAACTC TCTGTACCTG GCCACCATTT CTCTGAAGAT CGTGGCGTAC GTGAAGTATA ACTCCTCTCG TCCCAGAGAG GAGTGGGAGA TGTGGCACCC GACGCTAATA GCTGAGGCTT TGTTCGCCAT CGCCAACATC TTCAGCTCGC TCCGACTCAT CTCGCTCTTC ACCGCTAACT CCCACCTCGG GCCTCTGCAG ATCTCACTGG GGAGGATGCT GCTCGACATC CTCAAGTTTC TCTTCATTTA CTGTCTGGTC CTGCTGGCAT TCGCCAACGG TCTCAACCAG CTTTATTTCT ACTACGAGAC CAAGGCGTCA GAGGAACCCA ACAACTGTAA GGGCATCCGC TGCGAGAGGC AGAACAACGC ATTCTCAACG TTGTTTGAGA CTCTCCAGTC CCTCTTCTGG TCCATATTTG GGCTGCTGAA CCTCTATGTG ACCAACGTGA AGGCCCGCCA CGAGTTCACC GAGTTTGTGG GGGCGACCAT GTTTGGGACG TACAACATCA TCTCTCTGGT GGTTCTGCTG AACATGCTGA TCGCCATGAT GAACAACTCC TACCAGCTCA TTGCCGATCA CGCCGACATC GAGTGGAAGT TTGCGCGCAC CAAGCTGTGG ATGAGTTACT TCGACGAGGG CGGCACGCTG CCGCCCCCGT TCAACATCAT CCCCAGCCCC AAGTCCGTGT GGTACCTGCT GATGTGGCTT CACAACAGGC TGTGCCGCAG AGGACAGCCG CCCGGGGAGG AATCGCACAA ATGTGAAAAC CTGCGCGAGT TCACCGAGCG TCATGCTGAC AGCCTGATCC AGAATCAGCA TTACCAGGAA GTGATCCGGA ATCTGGTGAA GAGGTATGTG GCTGCCATGA TTCGCAGCGC CAAGACGGAC GAAGGGCTGA CGGAAGAAAA CTTCAAGGAA TTAAAACAAG ACATCTCTAG TTTTCGCTAC GAAGTTCTGG ATCTCCTGGG CAACCGGCGT CCTCCCAGAA GGAACTACTC CTCCTCCAGC GAGACGACAA AAGACGATGG CGCCATGGCA TCGGAAGATG ACAGTGAATT GGGTGATGGC GGCGAAGGAG GAGGAGGAGG GAGTGTGGGC ATCCAGAAGT CAAAGAGCGT CACCTTCACC AACCCAGTGG CGGACAACAG AAAGTCCACG CTTGGCGTGT CGGCGCTGGT GCGCTCCATA TCGGGAATGA CAAAGACTGA AAGAAAGGAT AAGGCGGAGC TGGAGGAGGG CAGTGGCTGG GGGGAGGAGG AGGAGGAAGA GGAGGGTAAA CCAAAGAGTA ACGGGCTGAA GACGACTGTC CTCCCTCCGT CTTCCACCTC TGTCCTCCCT TCGCCATCTT CATCAGTCTC CTCTTCGCTC ACGTCATCAC TGGCTCGCAC GAGGAGCCGG CTCCAGCGCC TCTCGGCTCC GAGCTCAAAG ACAGACTCTT TCAAACACCT CTCCTACTTG TTCTCTCGGT CCAAGCGCAG AGCCCCACCC ACGCCACTCC CCCTACAGTC CCCGCCCTCC TACACCATTT CTGACGGGTT GCTCTGCCCC TTAGGGAGCC ACAGCCACTC CGACTTTGGA CTCACCGATG TGACGAGAAG TGACACGCAC CTGAACGAGG TGGGCCGCTC GGTCGACAAC CCATCGTTCT CGCCTCGGAG AACGAATGGG CGAGACTCGC TGCTGACCCT GCCCCTCCCA CCCCCGTGCC CCTGCCAATC CCTGCACTGT GCTTCCAACA TGTCTGAGTC CAGCTCGCGC CTCCTGGACT CCAGCGAGGA CGTTTTCCTG GGTGGAGGCG GGGAAGGGGC GCTGGACGAC CACGGGGATG GAAGAGGGGG CATGCGGATG ATGTTGGGTG GGTGGGTGGG ACCCTGTGAT GATGTCATGG AGGACAGCTG  CGAGGTCATA GAGGACTCTG TCACAACGCA ACTATAG |

|  | **Genes** | **Forward, 5’ -> 3’** | **Reverse, 5’ -> 3’** |
| --- | --- | --- | --- |
| Zebrafish | *trpc5a* | GCTGCGAGAGGCAGAACAAT | AGGGTCAGGACTTCAAACCAGA |
|  | *trpc5b* | CTGCCACACAGCCTCCTATC | AGCTGTTGTACCTAGAACCCAA |
|  | *trpm2* | GAGCCGACCCTTGTGCAAAC | GTGCTGGACCTTCGGGTAAC |
|  | *trpm1b* | CAAGTACCTCGGACCCTACG | CCATAGCGTAAAGGTCTATCGAAT |
|  | *clcnk* | CACAAATCAGAGAGTGTGTGCT | CAGGTGGTGACCCTTCAGAC |
|  | *ano9b* | CGAATTGCAGGCGAATGGAG | AACCAGGGCACTGTGAACTC |
|  | *sp7* | AGGCGGGTCTCCTCTTTTCT | GGTCGCAGTTAGCATAGCCA |
|  | *actb1* | CGAGCAGGAGATGGGAACC | CAACGGAAACGCTCATTGC |
|  | *fgf23* | CATGCTGCATTCATCCGTCC | CTCGGGCTGATCTCCAGGTA |
|  | *sost* | ACGGACCAAACCGGACATAC | CTGCGGTGCTTCTTATTGCG |
|  | *sparc* | GCAGCTCCAACTGAAGAGGA | CTCCACGACCTCAATGGCTT |
| Medaka | *trpc5a* | GAGGTATGTGGCTGCCATGA | CGCCATCGTCTTTTGTCGTC |
|  | *trpc5b* | TTTTCTGAGTTCACGCCCGA | GATTTGATGTGGCCGAGGGA |
|  | *trpm2* | CCAGCCCCGAGGTTTTGTAT | TGGGCCACTTTGATGAGTCC |
|  | *trpm1b* | TATGGGCATGGAGAAGGGGATG | GTTGGATATGTCCTGGTGTGCT |
|  | *clcnk* | AGGGGTCAAAGGCCAAACAA | CACACTGCTCCCAAACAACG |
|  | *ano9b* | TTTGGACTAATGTGGGGGCG | TCAGCTCGCAGGCCATAAAA |
|  | *sp7* | CCGCATCTATTCTGGAGGTAGG | TCTGGTGTAGCCGAATCCCT |
|  | *actb1* | CCACCATGTACCCTGGCATT | GAGCCGCCAATCCATACAGA |
|  | *fgf23* | TTCTGTCCGGCAAGTAACCC | TTGACTCCAAGGATGGCGAC |
|  | *sost* | GCGAAAAACGGTGGAAGGAC | CCACCACTTTCCACGGATGA |
|  | *sparc* | GAGCTGGTCACAGAGGAACC | TCCTCCACAACCTCAATGGC |

**Supplementary table 2:** Predesigned primer sequences from Thermo Fisher Scientific for qPCR
